# Supplementary material for: Awareness of COVID-19 influences on the wellness of Thai health professional students: An ambulatory assessment during the early “new normal” informing policy
Source: PLoS One. 2021 Jun 14;16(6):e0252681. doi: 10.1371/journal.pone.0252681 (PMC8202936; doi:10.1371/journal.pone.0252681)
Supplement: S1 Table — (DOCX) [file pone.0252681.s001.docx]

**S1 Table. The score of the COVID-19 knowledge and practice tests.**

| Determinants (n = 1,001) | Median (min–max) | Mode (percentage) |
| --- | --- | --- |
| COVID-19 knowledge and practice tests (modified from the study of Bao-Liang Zhong et al., 2020 and the World Health Organization (WHO), 2020) (0–19) | 16 (2–19) | 17 (22.60) |
