# Supplementary material for: Awareness of COVID-19 influences on the wellness of Thai health professional students: An ambulatory assessment during the early “new normal” informing policy
Source: PLoS One. 2021 Jun 14;16(6):e0252681. doi: 10.1371/journal.pone.0252681 (PMC8202936; doi:10.1371/journal.pone.0252681)
Supplement: S2 Table — (DOCX) [file pone.0252681.s002.docx]

**S2 Table. Knowledge of COVID-19 influences on social well-being and quality of life among the Thai health professional students during the early “new normal” informing policy.**

| Variables  (n = 1,001) | R square | Coefficients  Std. Error | Standardized  Coefficient Beta | t | *p*-value |
| --- | --- | --- | --- | --- | --- |
| Dependent variable: Social well-being | 0.025 |  |  |  |  |
| Knowledge |  | 0.273 | 0.155 | 4.964 | < 0.001*** |
| Income |  | 0.000 | 0.026 | 0.835 | 0.404 |
| GPAX |  | 0.722 | -0.025 | -0.790 | 0.430 |
| Dependent variable: Quality of life | 0.022 |  |  |  |  |
| Knowledge |  | 0.310 | 0.139 | 4.431 | < 0.001*** |
| Income |  | 0.000 | 0.053 | 1.680 | 0.093 |
| GPAX |  | 0.820 | -0.025 | -0.806 | 0.420 |

*** *p*-value ≤ 0.001
