# Supplementary material for: Awareness of COVID-19 influences on the wellness of Thai health professional students: An ambulatory assessment during the early “new normal” informing policy
Source: PLoS One. 2021 Jun 14;16(6):e0252681. doi: 10.1371/journal.pone.0252681 (PMC8202936; doi:10.1371/journal.pone.0252681)
Supplement: S1 Raw data — (PDF) [file pone.0252681.s003.pdf]

S1 Raw data. Sociodemographic characteristics of the participants: Sex, program of study, year of study, GPAX, main source of income, income, feeling enough income, BMI, exercise behavior, smoking behavior, and alcoholic drinking behavior

No	Sex	Program	Year	GPAX	Source	Income	Feeling	BMI	Exercise	Smoking	Drinking
1	2	6	4	3	2	2	1	1	2	2	2
2	1	8	1	2	1	1	1	5	2	2	2
3	2	8	1	3	1	1	2	4	1	2	2
4	1	8	1	3	1	1	1	2	2	2	2
5	2	8	1	4	1	1	1	5	3	2	1
6	2	8	1	3	1	1	2	4	1	2	2
7	1	1	1	3	1	1	2	3	2	2	2
8	1	1	1	2	2	1	2	2	2	2	2
9	1	1	2	3	1	1	1	2	1	2	2
10	1	1	2	3	2	1	1	2	1	2	2
11	1	1	2	3	1	1	1	3	2	2	2
12	1	1	2	2	1	1	1	3	2	2	2
13	2	1	3	3	2	1	2	2	3	2	2
14	1	1	3	3	1	1	1	1	2	2	1
15	1	1	3	3	2	1	2	2	2	2	2
16	1	1	3	2	1	1	1	3	2	2	2
17	1	1	3	3	1	1	1	3	2	2	2
18	2	1	3	3	1	1	1	4	1	2	2
19	1	1	2	3	1	1	2	1	2	2	2
20	2	1	3	2	1	1	1	4	1	2	2
21	1	5	3	2	2	1	2	4	2	2	2
22	1	5	2	3	1	1	1	1	1	2	2
23	1	3	3	3	2	1	2	2	1	2	2
24	1	12	3	2	1	1	2	1	1	2	2
25	1	5	2	2	1	1	1	2	3	2	2
26	1	3	3	3	1	1	1	3	1	2	2
27	2	10	1	2	1	1	1	2	3	1	2
28	2	3	2	3	1	1	1	2	1	1	1
29	1	3	2	3	1	1	1	3	2	1	1
30	2	3	2	3	1	1	2	3	2	1	2
31	1	3	2	2	1	1	1	2	2	1	1
32	1	3	2	2	1	1	2	3	2	1	1
33	1	3	2	2	1	1	2	2	1	1	1
34	2	3	2	3	1	1	2	1	1	1	1
35	1	3	2	3	1	1	1	2	1	1	1
36	1	3	2	3	3	1	1	4	1	1	1
37	1	3	2	3	1	1	2	2	1	1	1
38	1	3	2	3	1	1	2	5	1	1	1
39	2	3	2	3	1	1	2	2	2	1	1
40	1	3	2	3	1	1	1	2	1	1	1
41	1	3	2	2	1	1	1	1	1	1	2
42	1	3	2	2	1	1	1	2	1	1	2

43	1	3	2	2	1	1	2	2	1	1	1
44	1	3	2	3	1	1	1	2	2	1	1
45	1	3	2	2	1	1	1	3	2	1	1
46	1	3	2	3	1	1	1	3	2	1	1
47	1	3	2	3	1	1	2	2	1	1	1
48	1	12	2	3	1	1	1	4	2	2	2
49	1	12	2	3	1	1	1	1	3	2	2
50	1	12	2	2	1	1	1	5	1	2	2
51	1	12	2	2	2	1	2	2	2	2	1
52	1	12	2	3	2	1	2	2	1	2	2
53	1	12	2	2	1	1	2	2	1	2	2
54	1	12	2	2	1	1	1	4	3	2	2
55	1	12	2	3	1	1	2	1	3	2	2
56	1	12	2	2	1	1	1	1	1	2	2
57	2	12	2	2	1	1	1	2	3	2	2
58	1	12	2	2	1	1	1	1	1	2	2
59	1	12	2	2	1	1	1	5	1	2	2
60	1	12	2	2	1	1	2	1	1	2	2
61	1	12	2	3	1	1	1	5	1	2	2
62	1	12	2	3	1	1	1	1	1	2	2
63	1	12	2	2	1	1	2	2	1	2	2
64	1	12	2	2	1	1	2	2	1	2	1
65	1	12	2	3	1	1	2	2	1	2	1
66	1	12	2	4	1	1	1	1	1	2	2
67	1	12	2	2	1	1	2	2	1	2	1
68	1	8	3	3	1	1	1	2	2	2	2
69	2	1	1	2	1	1	1	3	3	2	2
70	1	1	1	2	1	1	2	2	2	2	2
71	1	1	1	3	1	1	1	1	2	2	1
72	1	1	1	2	1	1	1	2	2	2	2
73	2	1	1	2	2	1	2	3	2	2	2
74	2	1	1	2	1	1	1	4	2	2	2
75	2	1	2	2	2	1	2	3	2	2	2
76	1	1	2	2	1	1	1	4	2	2	2
77	1	1	2	2	1	1	1	5	2	2	2
78	1	1	2	3	1	1	1	4	2	2	1
79	1	1	2	3	1	1	2	3	1	2	1
80	1	1	2	1	1	1	1	3	2	2	2
81	1	1	2	4	1	1	1	1	2	2	2
82	1	1	2	4	1	1	1	2	1	2	2
83	1	1	2	4	1	1	1	5	2	2	2
84	1	1	2	1	1	1	1	4	2	2	1
85	1	1	3	4	1	1	1	1	2	2	2
86	1	1	3	4	1	1	1	2	2	2	2
87	1	1	3	4	1	1	1	2	1	2	2
88	2	1	3	4	1	1	1	4	3	2	2
89	1	1	3	4	1	1	1	2	2	2	2

90	1	1	3	4	1	1	1	2	2	2	2
91	1	1	3	4	1	1	1	2	2	2	2
92	1	1	3	4	1	1	2	2	2	2	2
93	1	1	3	1	1	1	2	4	1	2	2
94	1	1	3	4	1	1	2	2	2	2	2
95	1	1	3	4	1	1	1	2	2	2	2
96	1	1	3	4	1	1	1	4	2	2	2
97	1	1	4	4	1	1	2	2	2	2	2
98	1	1	4	4	1	1	2	1	2	2	2
99	1	1	4	4	1	1	1	3	1	2	2
100	1	1	4	4	1	1	1	2	1	2	2
101	1	1	4	4	1	1	1	2	1	2	2
102	1	1	4	4	1	1	1	2	2	2	2
103	1	1	4	4	1	1	1	2	1	2	1
104	2	5	3	4	1	1	2	2	2	2	1
105	1	5	3	4	1	1	2	4	2	2	2
106	2	5	3	1	1	1	1	2	1	2	2
107	1	5	3	4	1	1	1	3	3	2	2
108	1	5	3	4	1	1	1	2	1	2	2
109	2	5	4	4	1	1	1	3	2	2	2
110	1	5	2	4	1	1	1	3	1	2	2
111	1	5	2	4	1	1	1	3	1	2	2
112	1	5	2	4	1	1	2	4	2	2	1
113	1	5	2	4	1	1	1	3	3	2	2
114	1	5	2	4	2	1	2	2	2	2	2
115	1	5	2	4	1	1	1	2	2	2	2
116	1	5	2	4	1	1	1	2	3	2	2
117	1	5	2	4	1	1	1	2	2	2	2
118	1	5	2	4	1	1	2	4	1	2	1
119	1	5	2	4	1	1	2	4	3	2	1
120	1	5	2	4	1	1	1	2	1	2	2
121	1	5	2	4	1	1	1	2	3	2	2
122	1	5	2	4	1	1	1	1	1	2	2
123	1	5	2	4	1	1	1	2	2	2	2
124	2	3	3	4	1	1	2	3	2	2	2
125	2	3	4	4	1	1	1	4	2	2	2
126	2	3	3	4	1	1	2	1	1	2	2
127	1	5	4	4	1	1	1	1	2	2	2
128	1	5	4	4	1	1	2	2	3	2	2
129	1	5	4	4	1	1	2	3	2	2	1
130	1	5	2	4	1	1	1	2	2	2	1
131	1	12	2	4	1	1	1	2	2	2	2
132	1	6	2	4	1	1	2	1	1	2	2
133	1	5	2	4	1	1	2	4	1	2	2
134	2	3	3	4	1	1	1	3	1	2	1
135	1	3	4	4	1	1	1	2	2	2	2
136	1	3	4	4	1	1	2	1	2	2	2

137	1	5	1	4	2	1	1	1	3	2	2
138	1	12	4	4	1	1	1	2	1	2	2
139	2	10	1	4	1	1	1	2	3	2	2
140	1	12	2	4	1	1	2	4	1	2	2
141	1	12	2	4	1	1	1	4	1	2	2
142	1	2	3	4	1	1	2	1	1	2	2
143	2	2	3	4	1	1	1	2	2	2	1
144	1	10	1	4	3	1	1	1	2	2	2
145	2	10	1	4	1	1	2	2	3	2	1
146	2	10	1	3	1	1	1	5	2	2	2
147	1	3	2	4	1	1	1	2	1	1	1
148	2	3	2	3	1	1	1	2	1	1	1
149	1	3	2	4	1	1	2	1	1	1	1
150	1	3	2	3	3	1	1	4	1	1	1
151	1	3	2	4	1	1	1	2	2	1	1
152	1	3	2	4	1	1	1	1	2	1	1
153	1	3	2	4	2	1	1	2	2	1	1
154	1	3	2	4	1	1	1	2	2	1	1
155	1	3	2	3	1	1	2	2	2	1	2
156	2	3	2	4	1	1	1	2	3	1	1
157	2	3	2	3	1	1	1	2	2	1	1
158	1	3	2	3	1	1	1	2	2	1	1
159	2	3	2	4	1	1	1	2	3	1	1
160	1	3	2	3	1	1	2	2	1	1	1
161	2	3	2	3	1	1	1	1	2	1	1
162	1	3	2	3	1	1	1	1	1	1	1
163	1	3	2	3	1	1	1	2	1	1	1
164	2	3	2	4	1	1	1	2	2	1	1
165	1	3	2	4	1	1	1	3	2	1	1
166	1	3	2	3	1	1	2	2	1	1	2
167	1	3	2	3	1	1	1	3	1	1	1
168	1	3	2	3	1	1	2	2	2	1	2
169	1	3	2	3	1	1	1	2	2	1	1
170	1	3	2	3	1	1	1	3	2	1	1
171	2	3	2	4	1	1	1	2	1	1	1
172	1	3	2	2	1	1	1	3	2	1	1
173	1	3	2	3	3	1	1	4	1	1	1
174	1	3	2	3	1	1	2	1	1	1	1
175	1	3	2	4	1	1	1	2	1	1	1
176	1	12	2	3	1	1	2	2	2	2	2
177	1	12	2	4	1	1	1	5	1	2	2
178	1	12	2	3	1	1	1	2	1	2	1
179	1	12	2	2	1	1	1	1	2	2	2
180	1	12	2	4	1	1	1	3	2	2	2
181	1	12	2	3	1	1	1	1	1	2	2
182	1	12	2	3	1	1	2	2	1	2	2
183	1	12	2	4	1	1	2	2	1	2	2

184	1	13	4	4	1	1	1	2	1	2	2
185	1	10	1	3	1	1	1	1	2	2	2
186	1	8	2	2	1	1	1	1	1	2	2
187	1	8	2	4	1	1	1	3	2	2	1
188	1	8	2	3	1	1	1	1	1	2	2
189	2	8	3	3	1	1	1	4	3	2	2
190	1	8	3	3	1	1	1	2	3	2	2
191	1	8	3	3	1	1	1	2	1	2	2
192	1	8	3	4	1	1	1	2	1	2	2
193	1	8	4	3	1	1	1	2	3	2	1
194	1	1	1	4	1	1	2	2	3	2	2
195	2	1	1	3	1	1	1	1	3	2	2
196	1	1	1	4	1	1	1	5	2	2	2
197	1	1	1	4	1	1	1	4	2	2	2
198	1	1	1	4	1	1	1	2	2	2	2
199	1	1	1	4	1	1	1	2	3	2	2
200	1	1	1	3	1	1	1	2	2	2	2
201	1	1	1	3	1	1	1	2	2	2	2
202	2	1	1	3	1	1	2	3	2	2	2
203	1	1	1	4	1	1	1	2	2	2	2
204	1	1	1	3	1	1	1	2	2	2	2
205	1	1	1	4	1	1	1	2	3	2	2
206	1	1	1	3	1	1	1	2	2	2	2
207	2	1	1	3	1	1	1	1	3	2	2
208	1	1	1	4	1	1	1	2	2	2	2
209	1	1	1	3	1	1	1	2	2	2	2
210	1	1	1	3	1	1	2	2	3	2	1
211	1	1	1	3	1	1	1	4	2	2	2
212	1	1	1	3	1	1	1	2	2	2	2
213	1	1	1	4	1	1	1	2	2	2	2
214	1	1	1	1	1	1	1	4	2	2	2
215	1	1	1	3	1	1	2	3	2	2	2
216	1	1	1	2	1	1	2	5	3	2	2
217	1	1	1	2	1	1	1	2	2	2	2
218	1	1	1	4	1	1	1	1	2	2	2
219	1	1	1	2	1	1	1	1	2	2	2
220	1	1	1	4	1	1	2	2	2	2	2
221	1	1	1	4	1	1	2	2	2	2	2
222	1	1	1	3	1	1	1	2	2	2	2
223	1	1	1	2	1	1	2	5	3	2	2
224	1	1	1	2	1	1	1	1	2	2	2
225	1	1	1	3	1	1	1	4	3	2	2
226	1	1	1	2	1	1	2	2	3	2	1
227	1	1	1	3	1	1	1	2	2	2	2
228	1	1	1	2	1	1	2	2	2	2	2
229	1	1	1	2	1	1	2	5	2	2	2
230	1	1	1	2	1	1	1	1	2	2	2

231	1	1	1	2	1	1	1	3	3	2	1
232	1	1	1	2	1	1	2	2	2	2	2
233	1	1	2	3	1	1	1	1	1	2	2
234	1	1	2	2	1	1	2	2	2	2	2
235	1	1	2	3	1	1	2	5	2	2	2
236	2	1	2	3	1	1	1	3	2	1	1
237	1	1	2	3	1	1	1	3	2	2	2
238	1	1	2	3	1	1	2	2	2	2	2
239	1	1	2	4	1	1	1	2	1	2	2
240	1	1	2	3	1	1	1	2	2	2	2
241	1	1	2	2	1	1	1	4	2	2	2
242	1	1	2	2	1	1	1	2	3	2	2
243	1	1	2	3	1	1	2	2	1	2	1
244	1	1	2	1	1	1	2	2	2	2	2
245	1	1	2	3	1	1	2	5	2	2	2
246	1	1	2	2	1	1	1	4	2	2	2
247	1	1	2	1	1	1	2	5	2	2	2
248	1	1	2	2	1	1	1	2	1	2	2
249	2	1	2	2	1	1	1	3	3	1	1
250	2	1	2	1	1	1	1	3	3	1	1
251	1	1	3	3	1	1	1	2	2	2	2
252	2	1	3	3	1	1	1	2	1	2	2
253	2	1	3	3	1	1	2	3	1	2	2
254	2	1	3	2	1	1	2	2	3	2	1
255	1	1	3	2	1	1	1	1	1	2	2
256	1	1	3	3	1	1	1	2	1	2	2
257	1	1	3	3	1	1	1	2	2	2	1
258	1	1	3	2	1	1	1	1	2	2	2
259	1	1	3	3	1	1	1	3	2	2	1
260	1	1	4	3	1	1	1	1	2	2	2
261	1	1	4	2	1	1	2	2	1	2	1
262	2	1	4	2	1	1	1	1	2	2	2
263	1	5	1	2	1	1	1	2	2	2	2
264	1	5	1	1	1	1	2	1	3	2	2
265	1	5	1	2	1	1	2	2	2	2	2
266	1	5	1	1	1	1	1	3	3	2	2
267	2	5	2	2	1	1	1	4	2	2	2
268	1	5	2	1	1	1	2	2	2	2	2
269	2	5	3	3	1	1	1	2	2	2	1
270	1	5	2	2	1	1	1	2	1	2	2
271	1	5	2	3	2	1	1	2	2	2	2
272	1	5	2	1	1	1	1	2	2	2	2
273	1	5	2	1	1	1	1	2	1	2	2
274	1	5	2	1	1	1	1	2	1	2	2
275	1	5	2	1	1	1	1	1	2	2	2
276	1	5	2	3	1	1	2	4	1	2	2
277	1	5	2	4	1	1	1	2	2	2	2

278	1	5	2	2	1	1	1	2	2	2	2
279	1	5	2	1	1	1	1	2	2	2	2
280	1	5	2	1	2	1	1	2	2	2	2
281	1	5	2	2	1	1	2	4	1	2	2
282	1	5	2	4	1	1	1	2	1	2	2
283	1	5	2	2	1	1	1	2	1	2	1
284	1	5	2	2	1	1	2	4	1	2	2
285	1	5	2	3	1	1	2	4	1	2	2
286	1	5	2	3	1	1	1	3	2	2	2
287	1	5	2	1	1	1	2	1	2	2	2
288	1	5	2	3	1	1	1	2	1	2	2
289	1	5	2	1	1	1	2	5	1	2	2
290	1	5	2	3	1	1	1	2	2	2	2
291	1	5	2	2	2	1	2	2	1	2	1
292	1	5	2	2	2	1	1	2	1	2	2
293	1	5	2	1	1	1	1	2	2	2	1
294	1	5	2	3	1	1	2	5	1	2	2
295	1	5	2	2	1	1	1	2	1	2	2
296	1	5	2	2	1	1	2	1	1	2	2
297	1	6	1	2	1	1	1	3	2	2	2
298	1	6	3	4	2	1	1	2	1	2	2
299	1	6	3	3	2	1	1	2	2	2	1
300	1	3	1	1	1	1	1	4	2	2	2
301	1	3	3	2	1	1	2	1	1	2	2
302	1	3	4	1	1	1	2	3	1	2	2
303	1	3	4	2	1	1	1	3	3	2	2
304	2	5	4	3	2	1	2	1	2	2	2
305	1	1	3	4	1	1	1	2	2	2	2
306	1	5	2	2	1	1	1	1	2	2	2
307	1	5	2	3	1	1	1	2	2	2	2
308	1	5	2	3	1	1	1	1	2	2	2
309	1	13	2	2	2	1	1	4	1	2	2
310	1	5	2	3	1	1	2	2	1	2	2
311	1	5	2	4	1	1	1	2	2	2	2
312	1	13	2	3	1	1	2	4	1	2	1
313	1	5	2	3	1	1	1	2	2	2	1
314	1	5	2	3	1	1	1	2	2	2	2
315	2	5	2	3	1	1	2	2	3	2	2
316	1	5	2	1	1	1	1	1	2	2	2
317	1	5	2	3	1	1	2	1	1	2	2
318	1	6	3	4	1	1	1	2	1	2	2
319	1	6	3	3	1	1	1	2	2	2	2
320	1	6	3	3	1	1	1	2	1	2	2
321	1	3	2	4	2	1	2	2	2	2	2
322	2	14	2	2	1	1	2	4	2	2	1
323	1	5	2	2	1	1	2	3	2	2	2
324	1	3	4	2	1	1	1	5	2	2	2

325	1	3	4	3	1	1	1	3	1	2	2
326	1	3	4	3	1	1	1	1	1	2	2
327	1	3	3	1	1	1	2	2	2	2	1
328	1	3	3	3	1	1	2	2	1	2	2
329	2	12	1	2	1	1	1	1	3	2	2
330	1	5	2	2	1	1	1	2	1	2	2
331	1	5	2	2	2	1	2	1	2	2	2
332	2	7	2	2	1	1	1	5	3	2	2
333	2	3	4	2	1	1	1	2	2	2	2
334	1	5	2	2	1	1	1	1	2	2	2
335	1	5	1	2	1	1	1	2	2	2	2
336	1	5	2	4	1	1	2	2	1	2	2
337	1	3	4	2	1	1	1	3	1	2	2
338	1	5	2	3	1	1	1	2	2	2	2
339	1	5	2	3	1	1	1	2	3	2	2
340	1	5	2	3	1	1	1	2	2	2	2
341	1	5	2	3	2	1	1	2	2	2	2
342	1	5	2	3	1	1	1	4	2	2	2
343	2	9	4	3	1	1	1	2	3	2	1
344	2	11	4	3	1	1	1	2	3	2	1
345	1	12	2	3	1	1	2	2	1	2	1
346	2	10	1	3	1	1	2	2	1	2	2
347	1	1	4	2	1	1	1	1	2	2	2
348	1	3	2	3	1	1	1	2	1	1	1
349	1	3	2	3	1	1	1	3	1	1	2
350	2	3	2	3	1	1	1	1	2	1	2
351	1	3	2	3	1	1	1	2	1	1	1
352	2	3	2	4	1	1	1	5	1	1	2
353	1	12	2	4	1	1	1	2	1	2	2
354	1	12	2	4	1	1	1	2	1	2	2
355	2	11	1	4	1	1	1	2	3	2	2
356	2	8	1	4	1	1	1	2	1	2	2
357	1	8	1	3	1	1	2	4	2	2	2
358	1	8	1	4	1	1	1	2	1	2	2
359	1	8	1	4	1	1	1	2	3	2	2
360	1	8	1	4	1	1	1	2	2	2	2
361	1	8	1	4	1	1	1	4	2	2	2
362	1	8	1	4	1	1	1	2	1	2	2
363	1	8	1	4	1	1	1	1	2	2	2
364	1	8	1	4	1	1	1	2	2	2	2
365	1	8	1	3	1	1	1	2	2	2	2
366	1	8	1	3	1	1	1	2	2	2	2
367	1	8	1	3	1	1	1	1	1	2	2
368	1	8	1	3	2	1	1	2	2	2	2
369	1	8	1	3	1	1	1	2	2	2	2
370	2	8	1	3	1	1	2	3	2	2	2
371	1	8	1	4	1	1	1	1	2	2	2

372	2	8	1	4	1	1	1	1	2	2	2
373	2	8	1	3	1	1	2	1	2	2	2
374	1	8	1	4	1	1	1	1	2	2	2
375	1	8	1	4	1	1	1	1	2	2	2
376	1	8	1	4	1	1	2	2	3	2	1
377	1	8	1	4	1	1	1	2	3	2	2
378	1	8	1	3	1	1	1	2	2	2	2
379	1	8	1	1	1	1	1	1	2	2	2
380	1	8	1	1	1	1	1	3	1	2	2
381	1	8	1	3	1	1	2	5	2	1	2
382	1	8	1	2	1	1	1	1	2	2	2
383	1	8	1	2	1	1	1	2	2	2	2
384	1	8	1	3	1	1	1	2	1	2	2
385	1	8	1	4	1	1	1	2	2	2	2
386	1	8	1	2	1	1	1	4	2	2	2
387	1	8	1	1	1	1	1	3	2	2	2
388	1	8	1	3	1	1	1	1	2	2	2
389	1	8	1	2	1	1	1	2	2	2	2
390	1	8	1	3	1	1	1	2	2	2	2
391	1	8	1	4	1	1	1	1	1	2	2
392	2	8	1	1	1	1	1	1	2	2	2
393	1	8	1	2	1	1	1	2	2	2	1
394	1	8	1	3	1	1	1	4	1	2	2
395	1	8	1	3	1	1	2	2	2	2	2
396	2	8	1	3	1	1	1	2	3	2	2
397	1	8	1	3	1	1	1	2	2	2	2
398	1	8	1	4	1	1	1	3	2	2	2
399	1	8	1	3	1	1	1	3	2	2	2
400	1	8	1	3	1	1	1	2	2	2	2
401	1	8	1	3	1	1	1	1	2	2	2
402	1	8	1	3	1	1	1	2	2	2	2
403	1	8	1	2	1	1	1	2	2	2	2
404	1	8	1	3	1	1	1	4	1	2	1
405	1	8	1	4	1	1	2	2	2	2	2
406	1	8	1	2	1	1	1	2	2	2	2
407	2	8	1	3	1	1	2	2	2	1	2
408	2	8	1	3	1	1	1	3	1	2	2
409	1	8	1	4	1	1	1	2	2	2	2
410	1	8	1	3	1	1	1	1	3	2	2
411	1	8	1	3	1	1	1	3	2	2	2
412	1	8	1	1	1	1	1	1	1	2	2
413	1	8	1	2	1	1	1	1	3	2	2
414	1	8	1	4	1	1	1	5	2	2	2
415	2	8	1	4	1	1	1	2	2	2	2
416	1	8	2	4	1	1	1	4	1	2	2
417	1	8	2	1	1	1	2	2	2	2	1
418	1	8	2	1	1	1	1	5	2	2	2

419	2	8	2	4	1	1	1	2	1	2	2
420	1	8	2	3	1	1	1	2	2	2	2
421	1	8	2	3	1	1	1	2	1	2	2
422	1	8	2	3	1	1	1	5	2	2	2
423	2	8	2	4	1	1	1	2	1	2	2
424	2	8	2	4	1	1	1	2	1	2	2
425	1	8	2	2	1	1	1	2	2	2	2
426	1	8	3	3	1	1	1	1	1	2	2
427	1	8	5	1	1	1	1	2	2	2	2
428	1	1	1	2	1	1	2	1	3	2	2
429	1	1	1	2	1	1	1	2	3	2	2
430	1	1	1	4	1	1	1	1	2	2	2
431	1	1	1	3	1	1	1	5	1	2	2
432	1	1	1	3	1	1	1	2	3	2	2
433	1	1	1	3	1	1	1	2	2	2	1
434	1	1	1	3	1	1	1	2	2	2	2
435	1	1	1	3	1	1	1	2	2	2	2
436	1	1	1	3	1	1	2	2	2	2	1
437	1	1	1	2	1	1	2	2	1	2	2
438	1	1	1	3	1	1	1	3	3	2	2
439	1	1	1	2	1	1	1	2	2	2	2
440	1	1	1	1	1	1	1	2	2	2	2
441	1	1	1	3	1	1	1	4	2	2	2
442	1	1	1	4	1	1	2	2	2	2	2
443	1	1	1	1	1	1	2	1	2	2	2
444	1	1	1	3	1	1	1	2	2	2	2
445	1	1	1	3	1	1	1	2	2	2	2
446	1	1	1	4	1	1	2	3	2	2	2
447	1	1	1	3	1	1	1	3	2	2	2
448	2	1	1	3	1	1	1	1	2	2	1
449	1	1	1	2	1	1	1	2	2	2	2
450	1	1	1	4	1	1	1	4	2	2	1
451	1	1	3	3	1	1	1	2	3	2	2
452	1	1	3	3	1	1	1	2	1	2	2
453	1	1	3	4	1	1	1	2	2	2	2
454	1	5	1	4	1	1	1	2	2	2	2
455	1	5	1	3	1	1	2	1	1	2	2
456	1	5	1	4	1	1	1	2	2	2	2
457	1	5	1	3	1	1	1	3	2	2	2
458	1	5	1	3	1	1	1	2	3	2	2
459	1	5	1	3	1	1	2	2	2	2	2
460	1	5	2	3	1	1	1	4	1	2	2
461	1	5	2	3	2	1	2	2	1	2	1
462	1	5	2	3	1	1	1	2	2	2	2
463	1	5	2	4	1	1	1	2	2	2	2
464	1	6	1	3	1	1	1	2	2	2	2
465	1	6	1	2	1	1	1	2	3	2	2

466	1	6	1	3	1	1	1	2	2	2	2
467	1	6	1	3	1	1	1	2	3	2	2
468	2	6	1	3	1	1	1	2	2	2	2
469	1	6	1	3	1	1	1	2	3	2	1
470	1	6	1	4	1	1	1	2	2	2	2
471	1	6	1	2	1	1	1	2	3	2	1
472	1	6	2	2	1	1	1	5	1	2	1
473	1	6	2	3	1	1	1	2	1	2	2
474	1	6	2	3	2	1	2	2	2	2	2
475	1	6	3	3	1	1	1	2	2	2	1
476	1	3	1	4	1	1	1	2	2	2	2
477	1	3	1	2	1	1	2	4	2	2	2
478	2	3	1	2	1	1	1	2	3	2	2
479	1	3	1	3	1	1	2	1	3	2	2
480	2	3	1	2	1	1	1	1	1	2	2
481	1	3	1	2	1	1	2	3	3	2	2
482	1	3	1	2	1	1	1	1	2	2	2
483	1	3	1	2	1	1	1	5	1	2	2
484	1	3	1	2	1	1	2	2	2	2	2
485	1	3	1	3	1	1	1	2	2	2	2
486	1	3	1	2	1	1	1	5	2	2	2
487	2	11	4	3	1	1	1	5	3	2	1
488	1	5	4	2	1	1	1	2	2	2	2
489	1	5	2	2	1	1	1	2	2	2	2
490	1	9	2	4	1	1	1	2	2	2	2
491	1	9	2	4	1	1	2	1	1	2	2
492	1	1	1	2	1	1	1	4	3	2	2
493	2	6	1	4	1	1	1	1	2	2	2
494	2	6	1	3	1	1	2	2	2	2	2
495	2	6	1	3	1	1	2	1	2	2	1
496	1	6	1	2	1	1	1	3	2	2	1
497	1	6	2	2	1	1	2	2	2	2	1
498	2	6	1	3	1	1	1	1	3	2	2
499	1	3	3	2	1	1	1	2	1	2	2
500	1	5	2	2	1	1	1	1	2	2	2
501	1	7	2	3	1	1	2	2	2	2	2
502	1	5	1	3	1	1	2	1	1	2	2
503	1	5	1	3	1	1	2	1	1	2	2
504	2	5	1	3	1	1	2	2	2	2	2
505	1	5	1	3	1	1	1	2	3	2	2
506	1	7	1	2	1	1	2	2	2	1	1
507	1	7	2	1	1	1	1	1	1	2	2
508	1	5	2	3	1	1	1	1	2	2	2
509	1	5	1	2	1	1	1	5	3	2	2
510	1	5	1	1	1	1	1	3	2	2	2
511	2	8	1	4	1	2	1	3	2	2	1
512	1	8	1	3	1	2	1	3	3	2	2

513	1	8	1	3	1	2	1	2	2	2	1
514	1	8	1	3	1	2	2	1	2	2	1
515	2	1	1	4	1	2	1	4	3	2	2
516	1	1	1	3	1	2	1	4	2	2	2
517	1	1	3	3	1	2	1	2	2	2	2
518	1	1	3	3	1	2	2	2	1	2	1
519	1	1	3	2	1	2	2	2	1	2	2
520	2	1	3	3	1	2	1	5	1	2	2
521	2	1	3	3	1	2	2	2	1	2	1
522	1	1	3	2	1	2	2	4	2	2	1
523	1	1	3	3	1	2	2	2	1	2	2
524	1	1	3	3	1	2	2	3	3	2	2
525	1	1	3	2	1	2	1	5	1	2	2
526	2	1	3	2	1	2	2	2	1	2	1
527	1	1	4	2	1	2	1	3	3	2	2
528	1	1	4	4	1	2	1	3	2	2	2
529	1	1	4	3	1	2	2	2	1	2	1
530	1	5	1	2	1	2	1	2	2	2	2
531	1	3	3	4	1	2	1	1	2	2	2
532	1	1	4	2	1	2	2	4	1	2	2
533	1	3	3	3	1	2	1	1	2	2	2
534	1	6	1	3	1	2	1	2	2	2	2
535	1	6	1	2	1	2	1	2	2	2	2
536	2	6	1	3	1	2	1	3	3	2	2
537	1	6	1	2	1	2	1	2	2	2	2
538	1	6	1	2	2	2	1	1	2	2	2
539	1	3	4	3	1	2	1	2	2	2	1
540	1	3	3	3	1	2	1	4	2	2	2
541	1	3	3	2	1	2	1	2	2	2	1
542	2	6	1	3	1	2	1	3	3	2	2
543	1	7	1	2	1	2	1	4	1	2	2
544	1	2	4	3	1	2	2	4	1	2	1
545	1	2	4	3	1	2	2	4	1	2	1
546	2	3	2	2	1	2	1	3	2	1	1
547	1	3	2	3	1	2	1	2	1	1	1
548	1	3	2	2	1	2	1	2	2	1	1
549	1	3	2	4	1	2	1	2	2	1	1
550	1	3	2	2	1	2	1	4	2	1	1
551	2	3	2	3	1	2	1	3	1	1	1
552	2	3	2	4	1	2	1	2	3	1	1
553	1	3	2	3	1	2	1	1	1	1	1
554	1	3	2	2	1	2	1	5	2	1	1
555	2	3	2	3	1	2	1	2	3	1	1
556	2	3	2	3	1	2	1	2	3	1	1
557	2	3	2	3	1	2	1	3	1	1	1
558	2	3	2	3	1	2	1	3	1	1	1
559	1	12	2	3	1	2	2	4	1	2	2

560	1	12	2	2	1	2	1	2	1	2	2
561	1	12	2	2	1	2	1	1	3	2	1
562	1	12	2	2	1	2	1	2	3	2	2
563	1	12	2	2	1	2	1	5	3	2	2
564	1	12	2	2	1	2	1	2	1	2	2
565	1	12	2	4	1	2	2	4	1	2	2
566	1	13	4	3	1	2	1	1	1	2	1
567	2	11	1	2	1	2	1	5	3	2	2
568	2	8	5	2	1	2	1	4	1	2	2
569	1	1	2	2	1	2	1	2	2	2	1
570	1	1	2	2	1	2	1	3	2	2	1
571	1	1	2	2	1	2	1	2	2	2	2
572	1	1	2	4	1	2	2	4	2	2	1
573	1	1	2	2	1	2	1	5	1	2	1
574	1	1	2	3	1	2	1	3	2	2	2
575	2	1	2	4	1	2	2	5	1	2	2
576	2	1	2	4	1	2	1	3	2	2	2
577	1	1	2	4	1	2	2	2	2	1	1
578	1	1	2	1	1	2	1	1	2	2	1
579	1	1	2	4	1	2	1	2	2	2	2
580	2	1	2	4	1	2	1	1	2	2	1
581	1	1	2	4	1	2	1	1	2	2	1
582	1	1	3	4	1	2	1	1	2	2	2
583	1	1	3	4	1	2	1	5	2	2	2
584	2	1	3	4	1	2	1	2	2	2	2
585	1	1	3	4	1	2	1	3	1	2	2
586	1	1	3	4	1	2	1	2	1	2	2
587	1	1	3	4	1	2	1	2	2	2	2
588	1	1	3	4	1	2	1	2	1	2	2
589	1	1	3	4	1	2	1	2	2	2	2
590	2	1	3	4	1	2	1	2	2	2	2
591	1	1	4	4	1	2	1	1	1	2	2
592	1	1	4	4	1	2	1	2	2	2	1
593	1	1	4	4	1	2	2	2	3	2	1
594	1	1	4	4	1	2	1	2	2	2	1
595	1	1	4	4	1	2	1	2	2	2	2
596	1	1	4	4	1	2	1	1	2	2	2
597	2	1	4	4	1	2	2	4	3	2	1
598	1	1	4	4	1	2	2	3	1	2	1
599	1	1	4	4	1	2	1	4	2	2	2
600	1	1	4	4	1	2	1	2	2	2	2
601	1	5	3	4	1	2	1	3	1	2	2
602	1	5	4	4	1	2	1	4	1	2	2
603	1	5	4	4	1	2	1	2	3	2	2
604	1	5	2	4	1	2	1	2	2	2	1
605	1	5	2	4	1	2	1	2	2	2	2
606	1	5	2	4	1	2	1	2	2	2	2

607	1	5	2	3	1	2	1	2	2	2	1
608	1	5	2	4	1	2	2	2	1	1	1
609	1	3	3	1	1	2	1	1	1	1	1
610	1	3	3	4	1	2	1	4	2	2	2
611	2	3	3	4	1	2	1	2	2	2	2
612	1	12	3	4	1	2	1	2	2	2	1
613	1	5	4	4	1	2	1	4	1	2	1
614	1	5	4	4	1	2	2	1	2	2	1
615	2	12	2	4	1	2	2	1	2	2	1
616	1	6	3	4	1	2	1	5	1	2	1
617	1	3	4	4	1	2	1	3	1	2	2
618	2	3	3	4	1	2	1	2	1	2	1
619	2	3	3	4	1	2	2	5	1	2	2
620	2	3	3	4	1	2	1	2	1	2	1
621	1	3	4	4	1	2	1	5	2	2	2
622	1	9	1	4	1	2	2	4	1	2	2
623	2	9	1	1	1	2	2	2	3	2	1
624	1	9	1	4	1	2	2	1	1	2	2
625	1	4	2	4	1	2	1	2	1	2	1
626	1	10	1	4	1	2	2	3	2	2	2
627	1	8	3	4	1	2	1	3	1	2	2
628	1	7	1	4	1	2	1	3	1	2	2
629	2	8	1	4	1	2	1	4	1	2	2
630	1	4	1	4	1	2	2	2	1	2	2
631	1	8	4	4	1	2	1	4	2	2	2
632	2	10	1	1	1	2	1	4	3	2	2
633	2	10	1	4	1	2	1	2	3	2	2
634	1	8	4	4	1	2	1	4	1	2	2
635	2	10	1	4	1	2	1	3	3	2	1
636	1	12	3	4	1	2	2	2	1	2	1
637	1	12	3	4	1	2	2	2	1	2	2
638	1	12	3	4	1	2	1	5	1	2	2
639	1	2	1	3	1	2	1	2	2	2	1
640	1	5	2	3	1	2	1	2	2	2	2
641	1	5	2	4	1	2	1	1	2	2	2
642	2	10	1	4	1	2	1	4	3	2	1
643	1	7	1	4	1	2	1	3	1	2	2
644	2	10	1	3	1	2	1	2	3	2	2
645	2	3	2	3	1	2	1	1	1	1	1
646	1	3	2	3	1	2	1	3	2	1	1
647	2	3	2	4	1	2	1	3	1	1	1
648	1	3	2	4	1	2	1	2	1	1	1
649	2	3	2	4	1	2	2	4	1	1	1
650	1	3	2	4	1	2	1	2	2	1	1
651	2	3	2	3	1	2	2	5	1	1	1
652	1	3	2	4	1	2	1	2	2	1	2
653	2	3	2	3	1	2	2	4	2	1	1

654	1	3	2	4	1	2	1	1	1	1	2
655	1	3	2	4	1	2	1	3	2	1	1
656	1	3	2	3	1	2	1	5	1	1	2
657	1	3	2	3	1	2	1	1	1	1	2
658	1	3	2	3	1	2	2	3	1	1	1
659	1	3	2	2	1	2	1	3	2	1	2
660	1	3	2	3	1	2	1	2	1	1	1
661	1	3	2	4	1	2	1	1	1	1	1
662	1	3	2	3	1	2	1	1	1	1	2
663	1	3	2	3	1	2	1	1	1	1	2
664	2	3	2	4	1	2	2	4	1	1	1
665	1	3	2	1	1	2	1	5	1	1	2
666	2	3	2	3	1	2	1	1	1	1	1
667	1	3	2	4	1	2	1	2	2	1	1
668	1	3	2	4	1	2	1	2	2	1	2
669	2	3	2	4	1	2	2	5	1	1	1
670	2	3	2	3	1	2	2	4	1	1	1
671	2	3	2	3	1	2	2	4	1	1	1
672	1	3	2	3	1	2	1	2	1	1	1
673	1	12	2	4	1	2	1	1	1	2	1
674	2	13	4	3	1	2	2	2	2	2	1
675	1	8	2	4	1	2	1	2	2	2	1
676	1	8	2	3	1	2	1	2	2	2	2
677	1	8	2	3	1	2	1	1	1	2	2
678	1	8	2	3	1	2	1	3	2	2	1
679	1	8	2	1	1	2	1	2	2	2	2
680	1	8	2	3	1	2	1	2	3	2	2
681	1	8	2	3	1	2	1	2	2	2	1
682	1	8	3	3	1	2	1	3	1	2	2
683	1	8	3	3	1	2	1	2	3	2	2
684	2	8	3	4	1	2	1	5	1	2	1
685	1	8	3	3	1	2	1	2	1	2	1
686	1	8	3	3	1	2	1	2	2	2	2
687	1	8	3	4	1	2	1	2	2	2	2
688	2	8	5	2	1	2	1	2	2	2	1
689	1	8	5	3	1	2	1	2	3	2	2
690	1	8	5	3	1	2	1	2	2	1	1
691	1	8	5	3	1	2	1	2	1	2	2
692	1	8	5	3	1	2	1	2	3	2	2
693	1	8	5	1	1	2	1	2	3	2	2
694	1	8	5	3	1	2	1	2	3	2	2
695	1	1	1	4	1	2	1	1	3	2	1
696	2	1	1	3	1	2	2	2	3	2	2
697	2	1	1	4	1	2	2	2	3	2	2
698	1	1	1	4	1	2	1	2	2	2	2
699	2	1	1	3	1	2	1	4	2	2	1
700	1	1	1	3	1	2	1	4	2	2	1

701	1	1	1	3	1	2	1	2	2	2	2
702	1	1	1	3	1	2	1	1	1	2	2
703	2	1	1	4	1	2	1	3	3	2	2
704	1	1	1	3	1	2	1	2	2	2	2
705	1	1	1	3	1	2	1	2	2	2	2
706	1	1	1	2	1	2	1	2	2	2	2
707	1	1	2	4	1	2	1	2	1	2	2
708	1	1	2	3	1	2	1	1	3	2	2
709	1	1	2	2	1	2	1	2	2	2	1
710	2	1	2	3	1	2	1	4	2	2	1
711	1	1	2	3	1	2	1	2	2	2	2
712	1	1	2	2	1	2	1	1	2	2	2
713	1	1	2	1	1	2	1	2	2	2	2
714	1	1	2	3	1	2	1	2	1	2	2
715	2	1	2	2	1	2	1	4	2	2	1
716	1	1	2	3	1	2	1	2	2	2	2
717	1	1	2	4	1	2	1	1	2	2	2
718	1	1	3	3	1	2	1	3	2	2	1
719	1	1	3	2	1	2	1	1	1	2	1
720	1	1	3	3	1	2	1	2	2	2	2
721	1	1	3	3	1	2	1	1	1	2	2
722	1	1	3	3	1	2	1	5	1	2	2
723	1	1	3	3	1	2	1	1	1	2	2
724	1	1	3	3	1	2	1	1	1	2	1
725	1	1	4	3	1	2	2	4	1	2	2
726	1	1	4	3	1	2	2	4	1	2	2
727	1	1	4	1	1	2	2	4	1	2	2
728	1	1	4	3	1	2	1	2	1	2	2
729	1	1	4	1	1	2	1	2	2	2	2
730	1	1	4	2	1	2	1	2	1	2	2
731	1	1	4	2	1	2	2	2	1	2	1
732	1	1	4	3	1	2	1	2	1	2	2
733	1	1	4	3	1	2	1	2	2	2	2
734	1	1	4	4	1	2	2	2	1	2	1
735	1	5	1	3	1	2	1	1	2	2	2
736	1	5	1	1	1	2	1	2	2	2	1
737	1	5	3	3	1	2	1	2	2	2	2
738	1	5	3	1	1	2	1	2	3	2	2
739	1	5	3	3	1	2	2	4	2	2	1
740	1	5	4	3	1	2	1	3	2	2	2
741	1	5	4	2	1	2	1	2	2	2	2
742	1	5	4	2	1	2	2	2	1	2	1
743	1	5	2	2	1	2	1	2	1	2	1
744	1	5	2	2	1	2	1	2	1	2	1
745	1	5	2	4	1	2	2	5	2	2	2
746	1	5	2	4	1	2	1	2	2	2	2
747	1	5	2	1	1	2	1	3	1	2	2

748	1	5	2	2	1	2	1	3	1	2	2
749	1	5	2	2	1	2	1	5	2	2	2
750	1	5	2	2	1	2	2	4	1	2	2
751	1	5	2	1	1	2	2	4	1	2	2
752	1	5	2	2	1	2	1	2	3	2	2
753	2	5	2	3	1	2	1	2	1	2	1
754	1	5	2	4	1	2	1	2	1	2	1
755	2	6	3	3	1	2	1	2	3	2	2
756	2	6	3	2	2	2	1	5	2	2	2
757	1	6	4	2	1	2	1	1	1	2	2
758	1	6	4	4	1	2	1	2	1	2	2
759	1	6	4	3	1	2	1	1	1	2	2
760	1	6	4	2	1	2	1	1	1	2	2
761	1	3	3	2	1	2	1	2	3	2	1
762	1	3	4	2	1	2	1	1	2	2	2
763	1	9	4	3	1	2	1	1	1	2	1
764	1	5	4	2	1	2	1	1	2	2	1
765	1	5	4	4	1	2	1	1	3	2	1
766	1	1	4	2	1	2	1	3	1	1	1
767	1	6	3	1	1	2	1	2	1	2	2
768	2	4	3	3	1	2	1	2	3	2	2
769	1	6	4	2	1	2	1	1	1	2	2
770	1	6	2	2	1	2	1	5	2	2	2
771	2	12	2	2	1	2	1	2	3	2	2
772	1	12	2	4	1	2	1	2	2	2	2
773	2	13	2	4	1	2	1	2	3	2	2
774	2	3	4	3	1	2	1	2	2	2	2
775	1	8	4	4	1	2	1	2	2	2	1
776	1	6	3	4	1	2	1	2	3	2	2
777	1	5	2	2	1	2	1	2	2	2	2
778	1	6	2	2	1	2	1	2	1	2	2
779	1	3	4	3	1	2	1	3	1	2	2
780	1	3	4	3	1	2	1	4	3	2	2
781	1	3	3	3	1	2	1	2	2	2	1
782	2	3	3	2	1	2	1	5	1	2	2
783	1	3	3	2	1	2	1	1	1	2	1
784	1	3	4	3	1	2	1	4	3	2	2
785	1	7	2	2	1	2	1	2	1	2	2
786	1	5	1	3	1	2	1	4	2	2	2
787	1	5	1	3	1	2	1	3	3	2	2
788	1	7	1	3	1	2	1	4	1	2	1
789	1	5	2	3	1	2	1	2	2	2	2
790	1	3	3	3	1	2	1	2	2	2	1
791	1	11	4	3	1	2	1	3	1	2	1
792	1	4	4	1	1	2	2	2	2	2	1
793	1	4	4	3	1	2	1	2	2	2	2
794	2	11	4	3	1	2	1	2	2	2	1

795	1	9	3	3	1	2	2	3	1	2	1
796	1	4	2	4	1	2	1	2	1	2	2
797	1	11	4	2	1	2	2	2	1	2	2
798	1	10	4	3	1	2	1	3	3	2	2
799	1	9	1	3	1	2	1	4	1	2	2
800	1	4	2	3	1	2	2	3	1	2	2
801	2	9	4	3	1	2	1	3	2	2	1
802	1	4	3	3	1	2	2	2	2	2	2
803	2	8	3	2	1	2	1	2	3	2	2
804	1	12	3	3	1	2	1	4	1	2	2
805	1	2	3	3	1	2	1	2	1	2	1
806	2	12	3	2	1	2	1	2	1	2	2
807	1	12	3	3	1	2	1	3	2	2	2
808	2	10	4	3	1	2	1	2	2	2	2
809	1	7	1	3	1	2	1	1	2	2	2
810	1	2	4	2	1	2	1	4	1	2	1
811	2	10	4	3	1	2	1	2	2	2	2
812	1	8	4	4	1	2	1	2	1	2	2
813	1	7	1	3	1	2	1	2	2	2	2
814	1	7	1	3	1	2	1	2	2	2	2
815	1	7	1	3	1	2	1	2	2	2	2
816	1	2	3	2	1	2	1	2	1	2	2
817	1	2	3	2	1	2	1	4	1	2	2
818	1	7	1	2	1	2	1	2	2	2	1
819	1	3	2	3	1	2	1	3	2	1	1
820	2	3	2	3	1	2	1	2	2	1	2
821	1	3	2	4	1	2	1	3	2	1	1
822	1	8	1	4	1	2	1	1	2	2	2
823	2	8	1	4	1	2	1	2	3	2	2
824	2	8	1	4	1	2	1	2	2	1	1
825	1	8	1	4	1	2	1	2	2	2	1
826	2	8	1	4	1	2	2	2	3	2	2
827	1	8	1	4	1	2	1	1	2	2	2
828	1	8	1	4	1	2	2	1	1	2	1
829	1	8	1	4	1	2	1	3	3	2	1
830	1	8	1	4	1	2	1	2	2	2	2
831	1	8	1	4	1	2	2	3	2	2	2
832	1	8	1	4	1	2	1	2	2	2	2
833	1	8	1	4	1	2	1	4	3	2	2
834	2	8	1	3	1	2	2	2	3	2	2
835	1	8	1	3	1	2	1	2	2	2	1
836	1	8	1	4	1	2	1	1	1	2	2
837	1	8	1	3	1	2	1	1	3	2	2
838	1	8	1	3	1	2	1	4	1	2	2
839	1	8	1	3	1	2	1	2	2	2	2
840	1	8	1	4	1	2	1	1	2	2	2
841	1	8	1	4	1	2	1	3	3	2	2

842	1	8	1	4	1	2	1	2	2	2	2
843	1	8	1	4	1	2	1	2	2	2	2
844	2	8	1	4	1	2	1	2	2	2	2
845	1	8	1	4	1	2	1	2	2	2	2
846	1	8	1	4	1	2	1	4	2	2	2
847	1	8	1	4	1	2	1	2	2	2	2
848	2	8	1	4	1	2	1	1	2	2	2
849	1	8	1	4	1	2	1	4	2	2	2
850	1	8	1	4	1	2	1	2	2	2	2
851	1	8	1	2	1	2	1	1	1	2	2
852	1	8	1	2	1	2	2	1	2	2	1
853	1	8	1	3	1	2	1	3	3	2	2
854	1	8	1	3	1	2	1	1	3	2	2
855	1	8	1	2	1	2	1	4	3	2	2
856	1	8	1	4	1	2	1	2	2	2	2
857	1	8	1	3	1	2	1	2	2	2	2
858	1	8	1	1	1	2	1	3	2	2	2
859	1	8	1	2	1	2	1	2	2	2	2
860	1	8	1	2	1	2	2	5	2	2	2
861	1	8	1	2	1	2	2	3	2	2	2
862	1	8	1	2	1	2	1	2	2	2	2
863	1	8	1	3	1	2	1	1	2	2	2
864	2	8	1	3	1	2	2	1	3	2	2
865	1	8	1	3	1	2	1	2	3	2	2
866	1	8	1	2	1	2	1	2	3	2	1
867	1	8	1	3	1	2	1	2	2	2	1
868	2	8	1	3	1	2	1	1	3	2	2
869	1	8	1	3	1	2	1	2	2	2	1
870	1	8	1	3	1	2	1	3	2	2	2
871	1	8	1	3	1	2	1	2	2	2	2
872	1	8	1	3	1	2	1	1	2	2	2
873	1	8	1	4	1	2	1	2	3	2	1
874	1	8	1	4	1	2	1	1	2	2	2
875	1	8	1	4	1	2	1	3	2	2	1
876	1	8	1	4	1	2	1	2	2	2	2
877	1	8	1	1	1	2	1	2	2	2	1
878	2	8	1	4	1	2	1	2	2	2	1
879	1	8	1	4	1	2	1	2	2	2	2
880	1	8	2	3	1	2	1	4	2	2	1
881	1	8	2	4	1	2	1	4	2	2	2
882	2	8	2	3	1	2	1	3	2	2	2
883	1	8	2	3	1	2	1	2	3	2	2
884	1	8	2	4	1	2	1	1	2	2	2
885	1	8	2	4	1	2	1	3	2	2	2
886	1	8	2	3	1	2	1	4	2	2	2
887	1	8	2	3	1	2	1	4	2	2	1
888	1	8	2	2	1	2	1	2	2	2	1

889	1	8	2	4	1	2	1	4	2	2	1
890	1	8	5	4	1	2	1	2	3	2	2
891	1	8	5	3	1	2	1	3	1	2	2
892	1	1	1	1	1	2	1	4	3	2	2
893	1	1	1	3	1	2	1	2	2	2	2
894	1	1	1	2	1	2	2	2	2	2	2
895	1	1	1	2	1	2	1	2	3	2	2
896	1	1	1	1	1	2	1	4	2	2	1
897	1	1	1	2	1	2	1	1	2	2	2
898	1	1	1	4	1	2	1	2	3	2	2
899	1	1	1	1	1	2	1	2	2	2	2
900	2	1	1	3	1	2	2	1	2	2	2
901	1	1	1	3	1	2	2	2	2	2	2
902	1	1	2	4	1	2	1	1	1	2	1
903	1	1	2	4	1	2	1	1	1	2	1
904	1	1	2	2	1	2	1	1	1	2	1
905	1	1	3	3	1	2	1	3	1	2	2
906	1	1	3	2	1	2	1	2	2	2	2
907	1	1	4	4	1	2	1	3	2	2	2
908	1	1	4	4	1	2	2	4	2	2	1
909	1	1	4	4	1	2	1	2	1	2	2
910	1	1	4	3	1	2	1	2	2	2	2
911	1	1	4	3	1	2	1	2	1	2	2
912	1	5	2	3	1	2	1	2	1	2	2
913	2	6	1	3	1	2	2	2	3	2	1
914	2	6	1	1	1	2	1	3	3	2	1
915	1	6	1	1	1	2	1	2	3	2	1
916	1	6	1	3	1	2	1	2	3	2	1
917	1	6	1	2	1	2	1	2	3	2	1
918	1	6	1	3	1	2	1	2	2	2	1
919	2	6	1	3	1	2	1	2	3	2	1
920	1	6	1	3	1	2	1	1	2	2	2
921	2	6	1	3	1	2	1	3	3	1	1
922	2	6	1	4	1	2	1	1	2	2	2
923	2	6	2	3	1	2	1	3	2	2	1
924	1	6	2	3	1	2	1	2	2	2	2
925	1	6	2	2	1	2	1	2	2	2	2
926	2	6	2	2	1	2	1	3	2	2	2
927	2	6	3	3	1	2	1	2	1	2	2
928	1	4	2	2	1	2	1	2	2	2	2
929	1	4	2	2	1	2	1	2	2	2	2
930	1	4	2	3	1	2	1	2	3	2	2
931	1	3	1	2	1	2	1	1	1	2	2
932	2	3	1	2	1	2	1	4	1	2	2
933	2	9	4	3	1	2	1	3	2	2	1
934	2	6	1	3	1	2	2	3	2	2	2
935	1	6	1	2	1	2	1	4	3	2	2

936	1	6	1	3	1	2	1	4	3	2	2
937	2	6	1	3	1	2	1	4	2	2	1
938	1	6	1	3	1	2	1	2	2	2	2
939	1	6	3	3	1	2	1	1	3	2	2
940	1	6	3	2	1	2	1	2	1	2	2
941	2	6	1	3	1	2	1	2	3	2	1
942	1	3	4	1	1	2	1	4	1	2	2
943	2	3	3	2	1	2	1	2	1	2	2
944	1	7	1	2	1	2	1	1	2	2	2
945	1	7	1	3	1	2	1	4	2	2	2
946	1	11	4	2	1	2	1	2	1	2	2
947	2	12	4	3	1	2	1	2	2	2	2
948	2	14	4	3	1	3	1	2	2	2	1
949	1	1	3	4	1	3	1	1	1	2	1
950	1	1	4	4	1	3	1	5	2	2	2
951	1	1	4	4	1	3	1	2	1	2	1
952	2	9	3	1	1	3	1	2	2	2	1
953	1	2	3	3	1	3	1	2	1	2	1
954	2	3	2	4	1	3	2	2	1	1	1
955	1	1	2	2	1	3	1	2	3	2	1
956	1	6	3	3	1	3	1	2	3	2	1
957	1	6	3	2	1	3	1	1	3	2	2
958	1	13	2	3	1	3	1	2	2	2	2
959	1	11	4	2	1	3	1	4	1	2	2
960	2	8	4	3	1	3	2	3	3	2	1
961	1	8	1	4	1	3	1	1	2	2	2
962	1	8	1	4	1	3	1	3	3	2	2
963	1	8	1	3	1	3	1	2	2	2	2
964	1	8	1	3	1	3	1	2	1	2	2
965	1	8	3	2	1	3	1	1	1	2	2
966	1	8	3	3	1	3	1	1	1	2	2
967	1	8	3	4	1	3	1	1	1	2	2
968	1	8	5	3	1	3	1	2	2	2	2
969	1	9	2	3	1	3	1	2	2	2	2
970	2	11	4	2	1	3	1	2	1	2	2
971	1	4	2	2	1	3	1	2	1	2	2
972	1	6	1	4	3	4	2	2	3	2	2
973	2	6	1	3	2	4	1	3	2	2	2
974	1	3	2	3	1	4	2	2	2	1	1
975	2	1	1	3	1	4	1	5	3	2	2
976	1	5	2	1	3	4	2	2	1	2	2
977	2	6	1	4	2	4	1	2	3	2	1
978	2	8	1	4	1	4	1	4	1	2	2
979	1	3	2	4	1	4	1	4	1	1	1
980	2	3	2	4	1	4	1	5	1	2	2
981	2	3	2	2	1	4	2	3	3	1	1
982	1	8	1	4	1	4	1	3	2	2	2

983	1	5	1	3	1	4	1	1	2	2	2
984	1	6	3	2	1	4	1	3	2	2	1
985	1	9	4	2	1	4	1	4	1	2	2
986	2	9	3	2	1	4	1	3	1	2	2
987	1	5	2	2	1	4	1	3	2	2	2
988	1	4	2	2	1	4	1	2	2	2	2
989	1	1	4	3	1	2	1	3	2	2	2
990	1	4	2	3	1	1	2	2	1	2	2
991	2	8	4	2	1	2	1	2	1	2	1
992	2	10	4	4	1	2	2	3	2	1	1
993	2	1	4	2	1	2	1	2	2	2	1
994	1	12	4	2	1	4	1	2	2	2	1
995	1	3	3	3	1	2	1	2	1	2	2
996	1	12	4	2	1	3	2	3	1	2	2
997	1	12	4	2	1	1	2	3	3	2	1
998	1	7	3	2	1	2	1	2	1	2	2
999	1	7	3	2	1	2	1	2	1	2	2
1000	1	12	4	2	1	1	2	4	2	2	2
1001	2	7	3	2	1	1	1	1	2	2	1

Sex: 1 is "female" and 2 is "male"; program: 1 is "physical therapy", 2 is "occupational therapy", 3 is "medical technology", 4 is "dental medicine", 5 is "nursing", 6 is "medecine", 7 is "Thai traditional medicine", 8 is "pharmacy", 9 is "sport science and exercise", 10 is "veterinary medicine", 11 to 13 is "public health", and 14 is "emergency medical operation" or "science and health technology"; GPAX: 1 is "< 2.50", 2 is "2.50–3.00", 3 is "3.01–3.50", and 4 is "3.51–4.00"; income: 1 is "< 5,000 (< 159.08)", 2 is "5,000–10,000 (159.08–318.17)", 3 is "10,001–15,000 (318.20–477.25)", and 4 is "> 15,000 (> 477.25)"; source: 1 is "parents or guardian", 2 is "scholarship", and 3 is "job"; feeling: 1 is "enough" and 2 is "not enough"; BMI: 1 is "underweight", 2 is "normal", 3 is "overweight", 4 is "obese I", and 5 is "obese II"; exercise: 1 is "not exercising", 2 is "exercising < 3 days/week", and 3 is "exercising ≥ 3 days/week"; smoking: 1 is "smoking" and 2 is "not smoking"; drinkink: 1 is "drinking alcoholic beverages" and 2 is "not drinking alcoholic beverages"
