# Supplementary material for: Awareness of COVID-19 influences on the wellness of Thai health professional students: An ambulatory assessment during the early “new normal” informing policy
Source: PLoS One. 2021 Jun 14;16(6):e0252681. doi: 10.1371/journal.pone.0252681 (PMC8202936; doi:10.1371/journal.pone.0252681)
Supplement: S2 Raw data — (PDF) [file pone.0252681.s004.pdf]

S2 Raw data. Self-report scales for COVID-19 awareness and preference of learning under the “new normal”, and the Stroop test score of the Thai health professional students_S1 The score of the COVID-19 knowledge and practice tests

No	Awareness	Aware_know	Aware_prac	Aware_att	Learning	Stroop	S1
1	29	10	10	9	9	20	18
2	26	7	10	9	8	18	19
3	26	7	9	10	8	19	16
4	24	9	6	9	8	20	19
5	28	10	10	8	6	20	18
6	21	7	8	6	2	19	13
7	20	3	8	9	7	18	16
8	18	6	6	6	10	2	2
9	18	6	7	5	4	19	16
10	19	9	2	8	8	18	14
11	29	10	10	9	8	19	14
12	26	10	8	8	5	19	16
13	24	8	8	8	6	20	18
14	25	9	9	7	7	20	16
15	30	10	10	10	10	19	18
16	20	9	3	8	6	18	16
17	26	8	9	9	10	20	18
18	26	8	9	9	7	18	18
19	16	5	6	5	5	19	16
20	26	10	8	8	10	5	16
21	28	8	10	10	10	20	16
22	23	6	10	7	5	19	16
23	30	10	10	10	5	20	17
24	27	10	9	8	10	18	16
25	25	8	8	9	7	12	18
26	24	7	9	8	7	20	17
27	28	10	8	10	5	18	17
28	14	7	2	5	8	19	18
29	24	7	10	7	10	19	17
30	16	2	8	6	5	12	18
31	18	5	7	6	8	20	18
32	23	8	8	7	5	19	19
33	21	9	2	10	8	19	16
34	23	8	7	8	8	19	19
35	25	8	9	8	5	19	17
36	19	10	1	8	9	19	15
37	24	6	9	9	8	20	16
38	29	9	10	10	9	20	7
39	19	10	1	8	7	19	18
40	13	4	4	5	4	19	15
41	28	8	10	10	7	17	16
42	25	9	8	8	7	19	18

43	25	10	10	5	10	20	18
44	27	10	10	7	1	18	18
45	21	7	7	7	10	19	15
46	25	9	9	7	7	20	19
47	29	9	10	10	6	19	18
48	25	8	9	8	5	20	16
49	15	8	2	5	6	19	19
50	21	7	10	4	4	19	14
51	29	10	10	9	10	19	14
52	25	8	9	8	9	19	19
53	25	8	9	8	7	20	17
54	22	8	8	6	7	19	17
55	27	9	9	9	8	19	15
56	25	7	9	9	9	19	17
57	28	9	10	9	9	19	19
58	25	9	9	7	8	19	19
59	27	9	10	8	10	20	18
60	17	5	6	6	8	10	14
61	24	8	8	8	6	20	14
62	23	7	8	8	9	18	18
63	26	9	10	7	1	20	18
64	26	8	9	9	5	20	14
65	25	8	9	8	7	19	15
66	28	10	8	10	6	20	19
67	23	6	8	9	5	19	16
68	18	9	1	8	8	20	19
69	23	8	8	7	7	19	18
70	19	7	7	5	1	20	18
71	24	8	8	8	9	20	15
72	24	7	9	8	8	20	18
73	17	5	4	8	3	19	11
74	23	8	7	8	8	19	18
75	30	10	10	10	5	19	16
76	29	10	10	9	9	20	17
77	25	10	10	5	10	20	18
78	25	8	9	8	5	20	16
79	23	6	10	7	5	19	18
80	24	6	9	9	8	20	16
81	26	8	9	9	10	20	16
82	23	8	8	7	5	19	18
83	25	8	8	9	7	12	13
84	25	8	9	8	7	19	15
85	26	7	9	10	8	19	17
86	25	8	9	8	7	19	15
87	30	10	10	10	10	19	18
88	20	9	3	8	6	18	16
89	25	10	10	5	10	20	18

90	28	8	10	10	7	17	16
91	20	9	3	8	6	18	17
92	21	7	8	6	2	19	16
93	25	8	9	8	5	20	16
94	24	7	9	8	7	20	16
95	14	7	2	5	8	19	19
96	28	10	8	10	5	18	18
97	29	10	10	9	8	19	14
98	26	7	10	9	8	18	19
99	25	8	8	9	7	12	13
100	25	8	8	9	7	12	13
101	28	9	10	9	9	19	13
102	25	9	8	8	7	19	13
103	25	9	8	8	7	19	18
104	19	6	7	6	10	20	16
105	16	3	7	6	3	3	16
106	26	8	9	9	7	17	15
107	17	10	1	6	8	20	18
108	24	8	9	7	9	17	17
109	21	7	7	7	6	19	16
110	28	9	10	9	5	19	16
111	24	8	8	8	4	19	16
112	23	7	8	8	4	17	17
113	28	9	10	9	7	19	15
114	13	6	1	6	6	19	19
115	26	9	10	7	8	19	15
116	20	10	2	8	5	20	18
117	21	6	8	7	5	10	16
118	25	8	8	9	7	19	17
119	20	6	9	5	8	19	17
120	16	8	2	6	7	19	16
121	20	5	10	5	6	20	16
122	20	5	10	5	5	19	16
123	25	9	8	8	7	19	15
124	21	8	7	6	6	20	19
125	15	6	1	8	3	19	15
126	25	7	9	9	8	15	18
127	24	9	6	9	5	19	17
128	19	6	7	6	4	19	17
129	28	9	10	9	5	19	13
130	19	5	7	7	6	19	13
131	24	8	9	7	9	17	13
132	24	7	9	8	8	20	13
133	26	9	10	7	6	19	17
134	19	6	7	6	10	20	16
135	24	9	6	9	5	19	16
136	19	6	7	6	4	19	15

137	15	5	5	5	5	19	18
138	21	10	1	10	7	19	17
139	21	10	1	10	7	19	16
140	24	9	6	9	5	19	16
141	21	10	1	10	7	19	16
142	18	8	2	8	6	5	16
143	20	8	4	8	3	19	18
144	26	8	10	8	10	20	18
145	22	7	10	5	1	17	17
146	15	8	4	3	4	13	14
147	20	9	1	10	7	10	18
148	28	10	10	8	8	18	15
149	28	10	9	9	9	10	16
150	18	7	5	6	6	19	16
151	19	8	8	3	2	19	16
152	26	10	8	8	7	19	14
153	18	6	6	6	6	20	18
154	22	8	7	7	5	20	15
155	20	5	5	10	5	20	18
156	29	10	10	9	2	18	18
157	17	6	5	6	5	15	15
158	26	8	9	9	1	18	18
159	17	4	5	8	6	19	13
160	22	7	7	8	5	20	18
161	30	10	10	10	10	20	18
162	30	10	10	10	5	16	15
163	28	9	9	10	10	20	17
164	30	10	10	10	1	20	16
165	28	9	9	10	8	20	17
166	29	10	10	9	7	20	17
167	27	9	9	9	5	20	15
168	18	8	1	9	9	20	12
169	24	8	10	6	1	19	17
170	30	10	10	10	8	19	17
171	18	8	3	7	6	16	17
172	25	7	10	8	7	19	17
173	24	9	10	5	8	19	15
174	30	10	10	10	5	20	17
175	22	7	8	7	2	19	19
176	27	8	10	9	5	20	18
177	23	7	8	8	5	19	14
178	25	8	9	8	6	19	18
179	17	8	6	3	3	20	16
180	16	7	4	5	5	19	15
181	20	7	6	7	7	19	15
182	28	9	9	10	7	19	18
183	24	8	9	7	8	20	16

184	18	8	2	8	3	20	19
185	28	9	9	10	2	19	18
186	21	7	7	7	6	19	18
187	22	8	8	6	7	19	15
188	26	7	10	9	4	20	18
189	20	7	6	7	10	19	17
190	22	6	8	8	3	19	16
191	18	7	1	10	6	19	16
192	23	8	10	5	7	20	16
193	24	8	8	8	9	20	11
194	28	10	8	10	5	19	18
195	20	8	9	3	7	20	15
196	26	9	9	8	9	19	18
197	27	9	9	9	6	20	17
198	20	8	7	5	7	19	16
199	19	4	8	7	6	18	13
200	30	10	10	10	7	19	13
201	21	6	8	7	5	20	13
202	28	10	10	8	7	20	13
203	18	10	2	6	3	20	18
204	13	6	6	1	5	20	12
205	25	8	9	8	8	18	16
206	28	10	10	8	1	19	15
207	25	9	9	7	7	18	16
208	30	10	10	10	3	19	17
209	13	9	1	3	3	20	16
210	15	5	5	5	5	19	14
211	30	10	10	10	5	18	16
212	25	6	10	9	5	19	17
213	17	10	1	6	3	19	14
214	24	8	9	7	4	19	19
215	24	9	10	5	8	18	15
216	17	8	1	8	5	19	17
217	17	9	3	5	1	20	17
218	27	9	9	9	7	19	17
219	15	6	4	5	3	20	13
220	26	8	10	8	2	20	16
221	27	10	9	8	5	12	16
222	22	8	8	6	4	20	15
223	18	10	1	7	4	19	15
224	30	10	10	10	5	19	19
225	18	2	7	9	6	19	15
226	21	5	9	7	5	19	17
227	25	9	8	8	6	18	17
228	30	10	10	10	10	19	17
229	16	5	6	5	1	19	13
230	27	9	9	9	8	18	13

231	24	8	8	8	8	20	13
232	21	10	1	10	3	19	13
233	30	10	10	10	10	18	18
234	23	9	6	8	3	19	14
235	20	6	8	6	7	18	12
236	14	8	3	3	8	19	15
237	18	5	6	7	5	19	18
238	30	10	10	10	4	18	17
239	25	8	9	8	2	19	16
240	20	6	5	9	8	19	16
241	21	6	6	9	5	20	16
242	15	5	7	3	5	18	16
243	22	8	7	7	1	19	15
244	20	7	4	9	8	20	17
245	24	9	10	5	8	18	17
246	24	8	9	7	3	9	18
247	20	10	1	9	5	19	18
248	27	8	9	10	1	19	15
249	18	6	5	7	4	19	14
250	21	6	8	7	5	20	16
251	28	9	9	10	8	19	16
252	23	8	6	9	8	20	15
253	20	9	6	5	6	19	16
254	28	9	9	10	6	19	15
255	22	7	7	8	7	20	17
256	18	6	6	6	3	5	18
257	24	8	9	7	3	19	16
258	27	9	9	9	3	19	18
259	21	6	6	9	5	20	17
260	28	9	10	9	9	20	18
261	28	10	10	8	1	20	17
262	16	5	6	5	1	19	17
263	20	8	2	10	2	19	18
264	24	8	9	7	5	19	16
265	20	9	3	8	9	1	13
266	22	7	9	6	2	19	16
267	27	8	9	10	1	19	14
268	28	10	8	10	5	19	12
269	22	8	7	7	7	20	16
270	18	5	6	7	5	19	17
271	26	8	9	9	2	20	16
272	21	5	9	7	5	19	16
273	15	5	5	5	5	8	15
274	21	7	9	5	10	19	16
275	24	7	9	8	7	19	18
276	26	10	8	8	10	19	18
277	20	9	2	9	7	20	13

278	22	6	9	7	7	19	18
279	29	9	10	10	4	20	16
280	22	6	8	8	3	19	18
281	20	7	6	7	10	3	18
282	25	8	9	8	6	11	18
283	25	7	9	9	1	2	16
284	26	8	9	9	10	12	18
285	25	9	9	7	9	15	19
286	24	7	9	8	7	14	16
287	21	7	7	7	2	10	17
288	17	5	6	6	6	12	18
289	26	9	9	8	8	14	16
290	18	6	7	5	4	14	15
291	25	8	8	9	8	15	16
292	25	10	10	5	1	14	13
293	28	9	10	9	8	15	13
294	27	8	10	9	9	10	18
295	29	10	10	9	9	9	16
296	27	8	9	10	10	14	18
297	18	8	3	7	4	12	17
298	20	5	7	8	8	13	16
299	25	8	8	9	9	12	16
300	25	8	8	9	1	15	18
301	23	8	7	8	7	15	18
302	24	7	9	8	7	14	9
303	28	10	9	9	5	13	14
304	25	8	9	8	6	11	19
305	17	8	2	7	7	15	19
306	25	8	9	8	6	11	15
307	25	10	10	5	1	14	12
308	26	9	9	8	8	14	6
309	26	9	9	8	8	14	15
310	19	6	5	8	6	13	16
311	26	9	9	8	7	8	16
312	23	7	8	8	7	15	16
313	30	10	10	10	4	13	18
314	25	8	10	7	8	15	16
315	30	10	10	10	10	13	18
316	25	8	10	7	8	15	17
317	29	10	10	9	9	4	18
318	23	9	7	7	7	12	18
319	25	8	10	7	8	15	16
320	26	9	8	9	8	12	16
321	24	7	9	8	5	10	16
322	26	10	9	7	10	13	15
323	26	10	9	7	10	13	17
324	27	9	9	9	9	10	16

325	13	4	5	4	1	15	17
326	30	10	10	10	5	19	17
327	20	9	2	9	8	15	18
328	24	8	8	8	5	14	16
329	25	9	9	7	5	15	16
330	26	8	10	8	8	15	18
331	28	10	9	9	10	14	17
332	12	7	3	2	7	15	17
333	24	7	9	8	5	14	16
334	20	6	7	7	6	14	19
335	30	10	10	10	10	14	19
336	24	7	9	8	5	14	15
337	24	7	9	8	5	14	16
338	24	7	9	8	2	14	7
339	21	7	7	7	8	20	18
340	18	7	6	5	7	20	16
341	21	9	6	6	10	20	18
342	23	7	7	9	8	20	17
343	20	9	2	9	8	20	18
344	24	8	8	8	7	19	18
345	21	8	8	5	3	16	15
346	21	7	8	6	5	20	19
347	24	7	9	8	6	20	19
348	22	7	7	8	8	20	15
349	30	10	10	10	3	20	19
350	26	10	9	7	3	20	10
351	27	9	9	9	7	20	14
352	19	10	1	8	6	20	19
353	24	8	9	7	8	20	16
354	18	7	2	9	5	19	17
355	22	8	7	7	1	17	11
356	18	7	2	9	5	19	16
357	26	9	9	8	1	19	19
358	26	10	10	6	9	20	19
359	26	9	9	8	8	20	18
360	19	8	2	9	8	16	14
361	25	8	8	9	6	20	11
362	14	6	3	5	7	19	15
363	23	6	9	8	6	19	16
364	13	5	3	5	5	19	17
365	19	1	8	10	7	20	17
366	22	10	2	10	6	20	19
367	22	8	7	7	1	20	17
368	22	10	7	5	5	20	19
369	20	7	8	5	3	17	16
370	13	9	1	3	4	20	17
371	20	5	9	6	6	20	16

372	22	10	2	10	8	20	17
373	28	8	10	10	2	16	16
374	29	10	10	9	6	19	18
375	24	8	8	8	9	20	16
376	25	8	8	9	8	19	16
377	27	8	10	9	6	20	18
378	21	6	7	8	4	20	14
379	22	6	8	8	1	20	18
380	24	8	9	7	9	20	16
381	27	9	9	9	5	20	16
382	30	10	10	10	1	20	18
383	28	10	10	8	8	20	12
384	25	7	9	9	7	20	17
385	21	5	8	8	3	20	15
386	18	6	7	5	3	17	16
387	20	3	9	8	8	20	17
388	21	8	4	9	8	19	17
389	20	9	1	10	8	17	18
390	22	5	8	9	6	20	18
391	26	9	9	8	7	20	18
392	21	10	1	10	6	20	17
393	30	10	10	10	10	20	16
394	30	10	10	10	9	20	16
395	23	9	10	4	1	20	19
396	13	9	1	3	4	20	18
397	19	7	6	6	6	20	15
398	13	6	3	4	6	17	19
399	27	9	9	9	6	17	18
400	24	8	7	9	7	16	16
401	26	9	8	9	1	17	17
402	26	8	10	8	8	20	15
403	21	6	9	6	3	13	17
404	19	7	6	6	8	20	16
405	29	10	9	10	10	19	15
406	20	7	6	7	8	20	16
407	25	8	9	8	9	20	17
408	26	8	10	8	8	20	16
409	19	5	7	7	3	20	17
410	25	8	9	8	9	20	18
411	16	6	2	8	5	20	16
412	15	7	3	5	7	20	15
413	19	8	2	9	2	20	16
414	24	8	8	8	5	20	19
415	30	10	10	10	9	20	16
416	22	6	9	7	6	17	19
417	30	10	10	10	10	13	16
418	24	8	8	8	7	20	15

419	17	5	5	7	3	19	17
420	18	5	6	7	5	16	18
421	23	7	10	6	7	20	14
422	16	5	6	5	5	20	11
423	16	5	8	3	1	20	15
424	28	9	10	9	4	13	18
425	26	8	9	9	7	15	16
426	21	7	7	7	1	15	17
427	16	5	5	6	2	20	16
428	28	10	10	8	6	19	14
429	21	6	7	8	8	20	18
430	29	10	9	10	1	10	17
431	24	8	8	8	3	19	16
432	27	9	9	9	5	18	18
433	17	8	1	8	5	19	15
434	21	7	7	7	1	20	14
435	27	9	9	9	1	20	15
436	19	7	7	5	5	20	16
437	27	8	10	9	6	20	17
438	25	9	10	6	6	20	19
439	18	4	8	6	2	20	16
440	30	10	10	10	8	20	16
441	27	8	10	9	5	17	15
442	12	8	2	2	9	20	18
443	22	8	7	7	3	19	17
444	26	10	10	6	3	20	17
445	20	6	7	7	5	20	16
446	27	10	8	9	6	20	17
447	17	6	5	6	7	20	14
448	24	7	9	8	7	20	16
449	21	6	7	8	4	19	14
450	29	9	10	10	10	17	17
451	21	7	8	6	3	20	17
452	25	7	9	9	7	19	16
453	24	8	9	7	8	20	18
454	17	8	2	7	8	17	14
455	21	7	7	7	7	20	16
456	23	9	8	6	5	19	16
457	13	5	3	5	4	19	18
458	21	10	1	10	5	19	17
459	27	7	10	10	1	20	15
460	20	6	9	5	3	13	11
461	26	6	10	10	4	10	15
462	22	7	8	7	7	19	13
463	26	8	10	8	9	20	19
464	21	7	9	5	6	20	16
465	21	10	2	9	8	20	17

466	21	6	8	7	7	20	17
467	23	8	8	7	8	20	14
468	19	8	3	8	3	20	19
469	26	8	9	9	5	19	16
470	15	4	1	10	7	13	17
471	19	8	2	9	2	20	17
472	27	9	9	9	9	20	18
473	21	7	8	6	9	19	17
474	19	8	2	9	2	20	17
475	13	5	3	5	4	19	18
476	21	10	1	10	4	19	18
477	21	10	2	9	8	20	17
478	24	8	9	7	9	20	15
479	26	10	10	6	3	20	17
480	30	10	10	10	8	20	16
481	12	8	2	2	9	20	16
482	21	7	7	7	7	20	16
483	27	9	9	9	9	20	15
484	25	9	10	6	6	20	18
485	21	10	2	9	8	20	17
486	21	6	7	8	4	19	14
487	19	8	3	8	3	20	19
488	27	9	9	9	9	20	15
489	25	9	10	6	6	20	18
490	27	7	10	10	1	20	15
491	25	7	9	9	7	19	17
492	25	7	9	9	7	19	16
493	26	8	9	9	5	19	16
494	21	9	2	10	9	19	18
495	18	6	6	6	7	20	17
496	30	10	10	10	6	19	18
497	20	10	2	8	5	19	16
498	21	9	2	10	8	20	17
499	25	8	9	8	6	20	18
500	26	8	9	9	7	20	15
501	24	7	9	8	9	18	14
502	23	7	8	8	10	20	17
503	23	7	9	7	6	19	17
504	17	6	6	5	8	19	18
505	18	8	4	6	7	19	16
506	27	8	9	10	8	19	15
507	27	9	9	9	7	19	17
508	21	7	8	6	6	18	19
509	21	10	1	10	10	19	17
510	24	9	10	5	1	18	16
511	22	8	8	6	7	19	17
512	26	9	8	9	6	19	18

513	26	9	9	8	8	20	13
514	23	8	8	7	3	19	15
515	22	7	8	7	9	20	17
516	22	7	9	6	8	19	17
517	30	10	10	10	5	19	17
518	28	8	10	10	8	19	15
519	27	9	10	8	6	19	18
520	17	6	8	3	5	19	17
521	24	8	8	8	9	20	17
522	21	10	1	10	1	19	17
523	27	10	10	7	4	19	16
524	29	9	10	10	6	19	18
525	29	9	10	10	6	19	17
526	30	10	10	10	10	19	17
527	29	9	10	10	5	20	16
528	24	7	9	8	9	15	15
529	22	6	8	8	9	19	18
530	26	8	9	9	4	20	14
531	25	8	9	8	7	19	15
532	25	8	9	8	4	19	18
533	23	8	8	7	1	18	16
534	30	10	10	10	3	19	18
535	19	8	4	7	6	19	17
536	13	7	1	5	1	19	15
537	27	10	8	9	10	18	17
538	25	8	9	8	10	19	19
539	17	6	6	5	8	19	17
540	23	7	8	8	9	19	18
541	27	9	9	9	5	19	18
542	27	9	10	8	7	17	18
543	27	9	9	9	9	19	17
544	21	8	8	5	3	19	14
545	14	6	3	5	1	19	18
546	25	9	9	7	6	19	19
547	22	8	8	6	7	19	18
548	25	8	9	8	10	19	18
549	21	10	1	10	1	19	16
550	25	8	9	8	7	19	17
551	22	7	8	7	9	19	17
552	19	10	1	8	1	20	12
553	26	9	8	9	6	19	17
554	26	9	9	8	8	19	13
555	30	10	10	10	4	20	16
556	24	5	10	9	2	20	16
557	19	10	1	8	6	19	18
558	27	9	9	9	10	18	16
559	22	10	3	9	6	19	15

560	28	10	9	9	7	19	15
561	24	8	8	8	5	20	16
562	27	9	9	9	10	19	15
563	24	8	7	9	5	18	17
564	28	10	10	8	3	19	17
565	27	9	9	9	10	19	16
566	27	8	10	9	4	17	17
567	14	8	1	5	8	19	17
568	18	8	1	9	6	20	17
569	16	7	3	6	5	19	15
570	25	10	10	5	7	20	16
571	14	5	2	7	4	20	16
572	16	5	1	10	6	20	19
573	28	10	9	9	7	19	17
574	21	6	9	6	3	19	18
575	25	10	8	7	8	19	17
576	21	7	5	9	5	20	14
577	19	8	1	10	10	20	18
578	27	9	9	9	8	19	17
579	24	9	8	7	6	19	17
580	27	9	10	8	5	19	17
581	18	8	2	8	5	19	15
582	17	5	9	3	1	18	17
583	19	7	9	3	7	20	12
584	19	8	2	9	3	19	18
585	21	10	3	8	5	19	17
586	16	7	3	6	5	19	15
587	26	9	9	8	6	20	16
588	30	10	10	10	8	19	18
589	28	10	10	8	7	19	15
590	17	9	1	7	5	19	16
591	27	9	9	9	8	19	15
592	19	8	3	8	4	19	16
593	19	4	7	8	2	19	12
594	30	10	10	10	5	20	18
595	27	9	10	8	7	20	17
596	26	9	10	7	3	19	19
597	24	9	8	7	9	19	16
598	25	6	10	9	4	18	16
599	23	8	7	8	8	19	14
600	30	10	10	10	5	15	17
601	19	7	3	9	7	20	17
602	27	10	9	8	6	19	17
603	21	9	2	10	9	20	16
604	30	10	10	10	9	18	17
605	21	8	8	5	5	17	16
606	20	9	2	9	5	20	18

607	24	8	8	8	5	17	17
608	28	8	10	10	8	17	17
609	22	3	9	10	3	15	19
610	26	8	9	9	5	16	19
611	17	7	2	8	4	17	19
612	22	7	7	8	5	20	19
613	20	8	2	10	10	20	19
614	30	10	10	10	5	20	17
615	16	5	1	10	5	20	16
616	20	6	7	7	6	17	17
617	27	9	9	9	8	17	18
618	25	8	10	7	3	17	19
619	25	8	9	8	8	20	19
620	27	10	9	8	10	20	19
621	25	10	8	7	9	17	17
622	26	9	9	8	7	17	19
623	27	10	9	8	5	17	19
624	27	9	10	8	7	20	16
625	26	9	9	8	6	17	17
626	24	8	8	8	5	20	17
627	25	9	10	6	8	15	18
628	27	9	9	9	1	17	18
629	30	10	10	10	10	19	17
630	25	9	8	8	7	19	17
631	23	9	8	6	4	19	17
632	25	8	8	9	10	16	17
633	25	10	10	5	2	19	17
634	21	6	8	7	5	19	17
635	21	9	3	9	8	20	16
636	27	9	9	9	8	18	13
637	25	5	10	10	10	19	17
638	30	10	10	10	1	19	17
639	21	5	8	8	9	18	18
640	28	10	8	10	5	20	17
641	28	10	8	10	5	20	17
642	18	10	1	7	6	19	18
643	26	9	8	9	6	20	17
644	27	9	9	9	10	5	17
645	25	8	9	8	6	16	14
646	27	9	10	8	4	19	15
647	26	9	9	8	6	19	17
648	27	9	10	8	4	19	15
649	27	9	9	9	8	15	18
650	17	5	6	6	5	19	17
651	27	10	10	7	10	20	17
652	17	6	6	5	8	18	17
653	25	7	9	9	10	19	14

654	24	8	9	7	7	19	17
655	19	10	1	8	8	18	17
656	26	8	9	9	8	19	16
657	26	10	9	7	8	19	17
658	22	7	9	6	6	19	18
659	25	8	8	9	3	19	17
660	23	8	8	7	6	18	19
661	24	8	8	8	9	19	19
662	27	7	10	10	4	10	17
663	24	8	8	8	8	19	19
664	25	8	8	9	3	19	17
665	24	7	8	9	8	10	16
666	30	10	10	10	10	19	15
667	18	8	2	8	7	19	15
668	28	10	10	8	4	19	16
669	23	7	7	9	6	19	15
670	23	7	7	9	6	19	15
671	21	8	7	6	6	19	16
672	24	9	7	8	6	18	14
673	30	10	10	10	10	19	16
674	26	9	8	9	6	17	16
675	27	9	9	9	8	18	18
676	22	9	8	5	8	18	17
677	28	9	9	10	7	19	19
678	23	8	8	7	6	18	15
679	19	8	2	9	7	19	17
680	23	8	7	8	9	19	18
681	25	7	9	9	10	19	18
682	27	9	9	9	8	19	17
683	26	8	9	9	7	20	16
684	30	10	10	10	8	19	16
685	27	8	10	9	5	19	19
686	28	9	9	10	7	19	19
687	27	9	10	8	8	19	16
688	22	8	7	7	1	18	19
689	21	8	8	5	8	20	18
690	17	5	6	6	5	19	17
691	22	9	8	5	8	18	17
692	21	8	6	7	7	19	16
693	18	8	3	7	9	19	15
694	26	8	9	9	7	19	16
695	22	8	7	7	5	19	17
696	24	8	8	8	7	20	17
697	23	8	8	7	8	19	18
698	21	7	7	7	4	20	16
699	20	7	5	8	6	20	14
700	18	8	2	8	5	20	17

701	23	8	7	8	2	20	18
702	18	7	8	3	7	20	16
703	24	8	7	9	6	17	17
704	19	7	3	9	6	17	13
705	20	7	7	6	6	19	17
706	20	7	5	8	6	20	14
707	24	8	8	8	4	16	13
708	23	5	8	10	4	20	16
709	12	5	5	2	5	16	15
710	25	10	8	7	4	20	16
711	21	8	6	7	2	20	17
712	21	8	6	7	3	20	17
713	23	8	6	9	3	20	13
714	23	8	6	9	3	20	13
715	23	8	6	9	3	1	11
716	21	7	7	7	6	12	18
717	18	10	1	7	4	20	15
718	25	8	9	8	4	17	17
719	24	8	8	8	6	20	13
720	25	8	9	8	10	20	18
721	21	8	8	5	3	20	16
722	25	8	9	8	8	19	13
723	28	10	10	8	8	20	17
724	26	9	9	8	9	20	17
725	26	8	9	9	8	19	15
726	27	9	8	10	6	20	18
727	19	8	3	8	6	20	17
728	30	10	10	10	7	20	15
729	19	8	3	8	6	20	17
730	30	10	10	10	1	17	16
731	15	8	3	4	4	20	17
732	18	8	5	5	4	20	10
733	15	8	3	4	4	20	17
734	18	10	3	5	1	18	14
735	19	6	5	8	6	19	16
736	24	9	7	8	1	20	16
737	25	10	9	6	7	20	16
738	29	10	10	9	10	19	18
739	24	8	8	8	9	19	18
740	25	10	10	5	7	20	18
741	16	7	3	6	3	20	16
742	25	9	7	9	7	19	18
743	21	5	8	8	2	19	18
744	18	8	2	8	7	7	18
745	18	8	4	6	5	20	13
746	19	8	6	5	1	19	16
747	24	8	8	8	5	20	18

748	18	9	2	7	6	18	17
749	25	8	9	8	6	19	18
750	17	8	2	7	3	20	18
751	30	10	10	10	5	19	18
752	23	8	8	7	5	20	15
753	16	5	9	2	2	20	18
754	23	10	3	10	3	20	17
755	26	9	9	8	7	18	15
756	24	8	8	8	8	20	16
757	22	10	2	10	6	20	18
758	30	10	10	10	4	20	15
759	23	6	9	8	7	19	18
760	22	10	2	10	4	17	13
761	30	10	10	10	1	16	17
762	13	6	4	3	7	20	16
763	27	8	10	9	10	19	18
764	25	7	10	8	3	12	18
765	24	8	8	8	7	19	15
766	24	10	10	4	6	19	18
767	22	7	8	7	7	19	17
768	27	9	9	9	8	19	14
769	19	10	2	7	9	19	16
770	26	10	8	8	8	20	18
771	18	9	2	7	7	19	18
772	24	9	10	5	8	18	16
773	20	8	6	6	9	15	15
774	16	10	1	5	3	20	18
775	24	8	9	7	9	19	16
776	19	8	3	8	7	20	17
777	20	8	8	4	5	20	15
778	26	8	8	10	5	19	17
779	18	8	2	8	2	19	17
780	28	10	10	8	10	20	17
781	30	10	10	10	3	19	18
782	17	7	3	7	7	18	15
783	17	8	1	8	8	19	16
784	27	8	10	9	7	19	19
785	29	10	10	9	8	19	14
786	24	8	8	8	6	19	19
787	17	6	5	6	5	18	14
788	20	9	2	9	10	20	17
789	16	9	1	6	8	19	17
790	15	6	3	6	4	19	18
791	26	10	10	6	1	19	16
792	25	7	10	8	8	19	17
793	25	8	9	8	9	19	19
794	23	7	9	7	10	18	16

795	24	9	8	7	8	19	15
796	23	8	7	8	5	20	17
797	30	10	10	10	5	18	15
798	14	8	1	5	4	18	17
799	26	8	9	9	9	18	18
800	27	9	9	9	8	19	15
801	13	9	2	2	2	16	16
802	18	8	3	7	8	19	17
803	16	9	1	6	8	19	17
804	19	7	7	5	5	18	18
805	15	7	3	5	5	19	15
806	25	9	8	8	8	19	15
807	17	6	1	10	8	19	17
808	19	6	6	7	6	19	14
809	21	10	1	10	10	17	16
810	19	6	8	5	3	18	17
811	30	10	10	10	6	20	17
812	26	8	10	8	8	13	11
813	21	6	8	7	5	19	16
814	23	7	8	8	9	19	17
815	18	5	6	7	9	20	15
816	20	6	7	7	4	19	18
817	26	9	9	8	8	20	19
818	14	8	1	5	7	19	17
819	18	10	5	3	8	20	12
820	17	9	2	6	2	19	15
821	20	8	3	9	6	19	17
822	9	5	1	3	4	20	15
823	19	8	3	8	7	19	16
824	19	7	8	4	1	19	14
825	24	8	8	8	3	20	19
826	16	6	3	7	6	19	17
827	24	8	8	8	5	19	19
828	26	10	9	7	6	19	18
829	24	8	8	8	6	18	18
830	24	9	9	6	10	19	14
831	16	10	1	5	10	20	16
832	15	7	1	7	1	20	18
833	27	8	10	9	7	18	17
834	25	7	10	8	10	19	17
835	15	9	3	3	10	20	18
836	19	8	2	9	6	18	17
837	29	9	10	10	7	19	17
838	12	5	2	5	4	19	13
839	30	10	10	10	10	8	16
840	24	8	9	7	8	2	16
841	21	10	1	10	8	19	16

842	21	8	8	5	6	20	16
843	18	1	10	7	7	20	18
844	26	10	9	7	4	17	17
845	20	8	3	9	6	20	18
846	21	8	8	5	3	17	16
847	19	3	8	8	1	19	18
848	19	4	8	7	2	19	9
849	24	7	9	8	6	19	16
850	30	10	10	10	6	19	17
851	24	9	10	5	1	20	16
852	19	10	1	8	7	19	16
853	30	10	10	10	7	20	17
854	26	9	9	8	6	19	16
855	28	8	10	10	2	19	14
856	21	7	7	7	10	16	16
857	19	9	2	8	9	19	17
858	24	8	8	8	3	20	17
859	23	7	8	8	10	19	15
860	14	2	6	6	4	19	18
861	30	10	10	10	9	19	18
862	27	9	9	9	6	18	11
863	24	9	8	7	7	19	18
864	19	9	1	9	8	17	15
865	28	9	10	9	10	20	17
866	26	8	9	9	9	20	11
867	20	8	3	9	7	17	18
868	27	8	9	10	4	20	17
869	25	9	8	8	5	19	14
870	26	9	9	8	8	17	14
871	16	5	5	6	7	19	14
872	30	10	10	10	7	20	18
873	19	8	2	9	7	19	15
874	22	7	8	7	7	16	19
875	27	9	9	9	8	19	17
876	26	9	9	8	3	19	18
877	30	10	10	10	9	19	16
878	28	9	10	9	8	19	8
879	19	1	10	8	6	19	13
880	18	6	5	7	5	20	19
881	26	8	9	9	9	20	19
882	27	10	9	8	10	20	13
883	23	8	8	7	7	19	15
884	21	8	7	6	6	1	15
885	22	7	9	6	9	18	17
886	16	4	7	5	1	19	18
887	23	8	8	7	8	20	15
888	19	5	7	7	4	19	17

889	27	9	9	9	8	18	17
890	20	8	6	6	4	19	18
891	30	10	10	10	5	19	18
892	19	7	7	5	8	19	17
893	18	8	1	9	4	20	17
894	20	9	1	10	9	19	17
895	22	10	2	10	7	19	16
896	30	10	10	10	10	17	14
897	28	8	10	10	2	19	14
898	20	9	1	10	7	18	17
899	23	7	8	8	5	19	18
900	18	6	7	5	6	20	15
901	20	9	2	9	6	19	17
902	26	8	9	9	5	19	18
903	27	10	10	7	4	19	18
904	20	8	3	9	6	19	19
905	30	10	10	10	7	20	16
906	14	2	6	6	4	19	18
907	22	8	7	7	5	20	16
908	26	7	9	10	10	17	17
909	23	7	9	7	4	17	17
910	24	8	9	7	5	18	17
911	28	8	10	10	4	18	16
912	24	9	8	7	5	20	14
913	25	9	10	6	6	18	17
914	23	8	7	8	7	16	13
915	21	8	8	5	3	18	13
916	29	10	10	9	10	20	16
917	20	7	6	7	6	20	13
918	21	7	7	7	5	18	17
919	22	8	8	6	1	17	11
920	21	7	8	6	5	18	15
921	26	9	8	9	8	19	15
922	18	6	6	6	5	18	13
923	17	6	6	5	5	19	12
924	20	7	7	6	4	19	13
925	24	9	9	6	8	19	15
926	17	6	6	5	5	17	11
927	16	5	6	5	1	18	14
928	17	5	7	5	6	20	12
929	21	7	7	7	5	18	18
930	21	7	7	7	9	15	11
931	16	5	5	6	3	20	11
932	17	6	6	5	6	17	13
933	14	5	5	4	4	20	11
934	18	6	6	6	3	20	15
935	16	5	6	5	3	18	11

936	14	5	4	5	1	20	12
937	16	6	6	4	4	20	10
938	17	6	6	5	9	20	12
939	15	5	5	5	5	19	9
940	21	7	7	7	6	19	15
941	19	7	7	5	5	19	14
942	18	7	6	5	5	17	10
943	22	8	6	8	6	19	19
944	24	9	9	6	8	20	15
945	11	4	4	3	6	19	8
946	21	7	8	6	6	20	13
947	20	8	6	6	7	15	12
948	20	7	7	6	5	18	9
949	18	6	6	6	5	20	19
950	13	4	5	4	5	18	11
951	21	10	10	1	1	20	17
952	21	5	8	8	6	20	18
953	23	10	7	6	3	18	14
954	25	9	8	8	7	20	14
955	22	7	8	7	7	18	13
956	21	7	7	7	5	18	11
957	21	7	6	8	4	18	19
958	14	4	4	6	2	20	12
959	19	6	7	6	5	16	12
960	16	6	6	4	7	20	16
961	18	6	6	6	5	20	18
962	25	7	9	9	5	19	16
963	29	9	10	10	9	19	12
964	19	6	7	6	5	16	12
965	21	7	8	6	3	17	11
966	20	7	6	7	5	14	12
967	21	8	6	7	5	20	13
968	21	8	7	6	4	17	15
969	18	6	6	6	5	19	11
970	19	5	7	7	3	19	15
971	20	7	8	5	5	20	16
972	23	8	6	9	3	19	15
973	21	9	6	6	5	20	19
974	24	7	8	9	10	18	13
975	21	9	6	6	5	20	19
976	22	7	9	6	7	20	16
977	26	7	9	10	4	19	13
978	25	7	9	9	4	19	14
979	16	6	6	4	2	14	15
980	18	7	5	6	6	19	16
981	18	6	6	6	5	19	14
982	27	9	8	10	9	9	18

983	25	8	9	8	9	19	9
984	17	6	3	8	6	20	17
985	25	8	9	8	6	19	17
986	25	8	8	9	9	20	15
987	23	10	8	5	9	6	9
988	18	6	6	6	5	19	12
989	21	8	5	8	7	19	13
990	20	8	7	5	6	20	11
991	23	10	8	5	8	19	16
992	22	8	9	5	6	19	15
993	26	9	10	7	4	19	17
994	25	7	9	9	7	19	19
995	24	7	9	8	2	19	16
996	20	10	2	8	5	17	15
997	21	10	1	10	5	19	17
998	19	6	6	7	3	18	16
999	20	6	6	8	7	19	16
1000	23	8	7	8	4	18	19
1001	20	7	7	6	8	20	18

Awareness is "COVID-19 awareness", aware_know is "COVID-19 knowledge", aware_prac is "COVID-19 protection practice", aware_att is "attitude towards COVID-19 pandemic", learning is "preference of learning under the “new normal”", Stroop is "Stroop test score", S1 is "COVID-19 knowledge and practice tests (modified from the study of Bao-Liang Zhong et al., 2020 and the World Health Organization (WHO), 2020)"
