# Supplementary material for: Awareness of COVID-19 influences on the wellness of Thai health professional students: An ambulatory assessment during the early “new normal” informing policy
Source: PLoS One. 2021 Jun 14;16(6):e0252681. doi: 10.1371/journal.pone.0252681 (PMC8202936; doi:10.1371/journal.pone.0252681)
Supplement: S3 Raw data — (PDF) [file pone.0252681.s005.pdf]

S3 Raw data. Physical activity, stress, mood, symptoms of anxiety and depression, social well-being, and quality of life among the Thai health professional students

No	IPAQ-SF	Walking MET	PSS-10	MDQ	GAD-7	PHQ-9	MSPSS	WHOQOL-BREF
1	3	160	2	1	1	2	80	2
2	3	254	2	1	1	4	71	2
3	3	0	1	1	1	1	75	3
4	3	0	1	1	1	1	75	3
5	3	0	1	1	1	2	72	2
6	3	280	2	1	2	3	63	2
7	3	175	2	1	1	1	60	2
8	3	0	1	1	1	1	36	1
9	2	0	2	1	1	1	84	3
10	3	120	2	1	1	1	54	3
11	3	278	2	1	2	4	65	2
12	3	0	2	1	2	4	60	2
13	3	0	2	1	1	1	84	3
14	3	0	2	1	1	2	48	2
15	3	0	3	1	1	4	72	2
16	3	250	1	1	1	1	78	2
17	3	0	2	1	1	1	60	2
18	2	250	2	1	1	1	36	2
19	3	0	2	1	1	4	54	2
20	3	0	1	1	1	2	72	3
21	3	40	2	1	1	1	84	3
22	3	250	3	1	1	3	60	2
23	1	273	2	1	1	3	68	2
24	3	0	2	2	1	2	72	2
25	3	275	2	1	1	1	47	1
26	2	262	2	1	1	1	60	3
27	3	0	2	1	1	3	71	2
28	3	0	2	1	1	2	48	2
29	3	265	2	1	1	1	83	3
30	3	120	2	1	1	3	24	1
31	3	60	3	1	1	4	48	3
32	3	0	3	1	1	4	36	2
33	3	0	1	1	1	1	24	3
34	3	0	1	1	1	1	12	1
35	3	0	2	1	1	2	71	3
36	2	0	2	1	1	3	69	2
37	3	0	2	1	1	1	84	3
38	3	0	1	1	1	1	60	2
39	3	130	2	1	1	1	84	3
40	3	0	1	1	1	1	59	2
41	3	240	2	1	1	3	67	2
42	3	0	2	1	1	3	69	2

43	2	252	2	1	1	1	78	3
44	3	0	1	1	1	1	69	3
45	3	40	1	1	1	4	67	2
46	3	280	2	1	1	1	71	3
47	3	0	3	1	3	1	52	2
48	2	80	2	1	1	1	58	2
49	3	40	2	1	1	4	76	3
50	3	0	2	2	1	4	78	2
51	3	240	1	1	1	2	81	3
52	3	0	1	2	1	4	49	2
53	3	240	2	1	2	3	42	2
54	3	0	2	1	1	4	39	2
55	3	48	2	1	1	4	75	2
56	1	168	2	2	1	4	67	2
57	3	0	2	1	1	1	72	2
58	3	0	2	1	1	1	82	2
59	3	0	2	1	1	2	69	2
60	3	48	2	1	1	1	30	2
61	3	0	2	1	1	1	61	2
62	3	160	1	1	1	2	47	2
63	3	0	2	1	1	1	63	2
64	3	236	2	1	3	3	65	2
65	3	240	2	1	1	1	69	3
66	3	0	2	1	1	2	60	2
67	2	235	2	1	1	1	76	3
68	2	236	2	1	1	4	61	2
69	3	80	2	1	1	1	72	3
70	3	244	2	1	1	2	77	2
71	3	252	2	1	1	4	59	2
72	3	0	2	1	1	1	70	2
73	3	0	2	1	1	4	54	2
74	3	0	2	1	1	3	84	2
75	3	0	2	1	1	4	69	2
76	3	260	2	1	1	1	80	2
77	2	252	2	1	1	1	78	3
78	3	80	2	1	1	1	58	2
79	3	243	3	1	1	3	60	2
80	3	0	2	1	1	1	84	3
81	3	0	2	1	1	1	60	2
82	3	0	3	1	1	4	36	2
83	3	280	2	1	1	1	47	1
84	3	240	2	1	1	1	73	3
85	3	0	1	1	1	1	75	3
86	3	240	2	1	1	1	70	3
87	3	0	2	1	1	4	72	2
88	3	223	1	1	1	1	78	2
89	2	252	2	1	1	1	78	3

90	3	269	2	1	1	3	67	2
91	3	260	1	1	1	1	77	2
92	3	280	2	1	2	3	63	2
93	2	80	2	1	1	1	58	2
94	3	240	2	1	1	1	60	3
95	3	0	2	1	1	2	48	2
96	3	0	2	1	1	3	69	2
97	3	240	2	1	2	4	63	2
98	3	240	2	1	1	4	71	2
99	3	280	2	1	1	1	47	1
100	3	275	2	1	1	1	47	1
101	3	0	2	1	1	1	72	2
102	3	0	2	1	1	3	69	2
103	3	0	2	1	1	3	69	2
104	3	0	1	1	1	1	84	2
105	2	230	2	2	1	1	32	2
106	2	160	2	1	1	2	72	3
107	3	0	1	1	1	1	80	3
108	3	120	2	1	2	3	71	2
109	3	0	2	1	1	4	63	2
110	3	280	1	2	1	3	71	3
111	1	270	1	2	1	3	46	2
112	3	0	2	1	2	4	67	2
113	3	120	2	1	2	4	51	2
114	2	160	2	1	1	4	74	2
115	3	0	2	1	1	1	76	3
116	3	0	2	1	1	2	76	2
117	3	0	2	1	1	4	68	2
118	3	0	2	1	1	3	48	2
119	3	0	2	1	1	2	48	2
120	3	0	2	1	1	1	76	3
121	3	0	1	1	1	1	77	2
122	3	180	1	1	1	1	77	2
123	2	173	2	1	1	2	72	2
124	3	0	2	1	1	1	58	2
125	3	0	2	1	1	3	61	2
126	3	0	2	1	1	1	72	3
127	3	0	1	1	1	1	74	2
128	3	180	2	1	1	2	63	2
129	3	275	1	1	1	2	71	3
130	3	140	2	1	1	3	76	3
131	3	120	2	1	2	3	71	2
132	3	0	2	1	1	1	70	2
133	3	0	3	1	1	1	62	2
134	3	0	1	1	1	1	84	2
135	3	0	1	1	1	1	74	2
136	3	280	3	1	1	2	63	2

137	3	0	2	1	1	1	72	3
138	3	240	2	1	1	1	74	3
139	3	240	2	1	1	1	71	3
140	3	0	1	1	1	1	74	2
141	3	240	2	1	1	1	74	3
142	3	240	2	1	1	1	74	2
143	3	0	2	1	1	2	76	3
144	3	240	2	1	1	2	74	2
145	3	0	2	1	1	1	84	3
146	2	0	2	1	1	1	70	2
147	2	0	2	1	1	2	58	3
148	1	0	2	1	1	1	83	3
149	3	0	3	1	1	4	24	1
150	3	160	2	1	1	1	29	2
151	2	168	2	1	1	2	68	2
152	3	0	2	1	1	2	51	2
153	1	260	2	1	3	4	36	2
154	3	280	2	1	1	3	60	2
155	3	268	2	1	1	4	82	3
156	1	280	2	1	1	1	84	3
157	2	0	2	1	1	2	60	3
158	3	180	2	1	1	1	68	3
159	1	280	2	1	1	2	72	2
160	3	0	2	1	1	1	84	2
161	2	260	2	1	1	1	84	3
162	2	275	1	1	1	1	72	3
163	3	0	2	1	1	1	67	3
164	1	240	2	1	1	2	84	2
165	2	220	2	1	1	1	74	2
166	2	240	1	1	1	1	72	3
167	1	240	2	1	1	2	83	3
168	3	264	1	1	1	1	43	3
169	3	248	2	1	3	4	75	2
170	2	260	2	1	1	3	84	3
171	2	160	2	1	1	1	68	3
172	1	260	1	1	1	1	45	3
173	2	240	1	1	1	1	82	3
174	1	0	2	1	1	1	72	3
175	2	260	2	1	1	2	62	2
176	3	40	2	1	1	1	62	3
177	3	196	2	1	1	2	62	2
178	3	240	2	1	1	1	70	2
179	3	0	2	1	1	3	68	2
180	3	0	2	1	1	1	75	2
181	3	240	1	1	1	1	53	2
182	2	280	2	1	1	1	81	3
183	3	0	2	1	1	4	67	2

184	2	248	1	1	1	1	82	3
185	2	250	2	1	1	1	62	2
186	2	268	2	1	1	1	72	2
187	3	240	1	1	1	1	72	3
188	2	0	2	1	1	1	74	3
189	3	0	2	1	1	1	78	3
190	2	240	2	2	2	4	50	2
191	3	240	2	1	1	2	79	3
192	2	265	2	1	1	3	76	3
193	3	0	2	1	2	4	49	1
194	3	0	3	1	1	1	36	1
195	1	236	2	1	1	1	84	3
196	2	0	2	1	1	4	48	1
197	2	200	2	1	1	1	65	3
198	2	260	2	1	1	2	67	1
199	3	0	2	1	1	2	70	1
200	2	268	2	1	1	1	84	3
201	2	144	1	1	1	1	48	1
202	2	280	2	1	1	1	81	3
203	1	136	2	1	2	4	61	3
204	1	0	2	1	1	2	40	1
205	1	268	1	1	1	1	70	3
206	2	0	1	1	1	1	72	3
207	2	260	2	1	1	1	80	3
208	3	280	2	1	1	1	77	3
209	3	0	2	1	1	1	38	2
210	3	160	2	1	1	1	53	1
211	3	0	1	1	1	1	84	3
212	3	0	2	1	1	1	65	2
213	2	260	2	1	1	4	74	2
214	3	0	1	1	1	2	68	2
215	1	280	1	1	1	1	62	3
216	2	220	2	1	1	1	67	2
217	2	280	2	1	1	3	49	2
218	3	120	2	1	2	4	83	2
219	3	0	2	1	1	4	59	2
220	2	270	2	1	1	1	80	2
221	3	0	2	1	1	1	84	3
222	2	80	2	1	1	2	67	2
223	3	0	3	1	2	2	81	3
224	2	260	2	1	1	1	67	2
225	2	0	2	1	1	1	67	2
226	2	260	2	1	1	3	73	2
227	2	180	2	1	1	1	71	3
228	3	0	2	1	1	1	58	2
229	3	0	2	1	1	2	50	2
230	3	0	2	1	1	2	48	2

231	3	0	1	1	1	1	84	2
232	3	260	2	1	1	1	70	3
233	3	248	1	1	1	1	82	3
234	3	0	2	1	1	2	71	2
235	3	230	2	1	1	1	69	2
236	3	0	2	1	1	1	65	3
237	3	0	2	1	1	4	62	2
238	3	260	2	1	1	1	78	3
239	3	0	2	1	1	1	77	2
240	3	0	2	1	1	1	57	2
241	3	0	2	1	1	1	71	2
242	3	100	2	1	1	1	75	3
243	3	200	2	1	1	4	62	3
244	3	80	2	1	1	1	68	2
245	3	260	2	1	1	3	71	2
246	3	0	2	1	1	1	47	2
247	2	192	2	1	1	2	68	2
248	2	240	2	1	1	1	79	2
249	3	192	2	1	2	4	50	2
250	2	144	1	1	1	1	48	2
251	2	230	2	1	1	1	72	2
252	1	280	2	1	1	4	48	2
253	3	40	2	1	1	3	62	2
254	3	240	2	1	1	2	68	2
255	1	240	1	1	1	1	62	2
256	3	270	1	2	1	2	68	1
257	3	0	2	1	1	2	52	2
258	3	40	1	1	1	1	72	2
259	3	0	2	1	1	1	72	1
260	1	248	2	1	1	4	66	3
261	3	0	2	1	1	1	76	3
262	3	0	2	2	1	2	50	2
263	2	230	2	1	1	1	81	3
264	3	210	2	1	1	1	72	3
265	3	0	2	1	1	2	58	1
266	3	0	2	1	1	1	73	3
267	2	240	2	1	1	1	79	3
268	3	0	3	1	1	1	36	2
269	2	240	2	1	1	1	59	2
270	3	0	2	1	1	4	60	2
271	2	0	1	1	1	2	75	2
272	2	260	2	1	1	3	73	2
273	3	0	1	1	1	3	47	1
274	3	260	2	1	1	4	71	3
275	1	268	2	1	1	2	79	2
276	3	0	1	1	1	1	72	2
277	1	0	2	1	1	1	71	3

278	2	236	2	1	1	4	84	3
279	2	240	2	1	1	1	64	3
280	3	280	2	2	2	4	50	2
281	2	275	2	1	1	1	48	3
282	2	0	3	2	2	4	73	2
283	3	278	2	1	1	1	56	3
284	3	0	2	1	1	1	82	3
285	2	268	2	1	1	2	81	2
286	3	0	1	1	1	1	72	2
287	2	120	2	1	1	1	48	2
288	3	0	1	1	1	2	12	2
289	3	200	1	1	1	1	67	3
290	3	0	2	1	1	2	51	2
291	3	0	2	1	1	1	74	3
292	3	0	2	1	1	3	71	2
293	2	280	2	1	1	2	78	3
294	3	0	2	1	1	1	84	3
295	3	0	1	1	1	1	84	3
296	3	0	2	1	1	1	48	3
297	3	0	2	1	1	1	72	3
298	3	0	1	1	1	1	75	3
299	3	0	2	1	1	2	71	3
300	3	0	3	1	1	1	48	2
301	3	0	2	1	1	3	72	2
302	3	0	2	1	1	3	48	2
303	2	280	2	1	1	1	79	3
304	2	0	3	2	3	4	70	2
305	3	0	2	1	1	1	64	3
306	2	0	3	2	3	4	73	2
307	3	0	2	1	1	4	71	2
308	3	200	1	1	1	1	71	3
309	3	200	1	1	1	1	71	3
310	3	240	2	1	1	1	60	2
311	1	0	1	1	1	1	72	2
312	3	0	1	1	1	1	48	2
313	3	0	3	1	2	4	55	2
314	3	240	2	1	2	2	61	2
315	1	240	2	1	1	1	84	3
316	3	240	2	1	2	2	61	2
317	3	0	1	1	1	1	72	3
318	3	0	2	1	1	2	42	2
319	3	240	2	1	2	2	61	2
320	2	280	2	1	1	1	70	2
321	3	0	2	1	1	3	36	2
322	2	0	1	1	1	1	84	2
323	2	0	1	1	1	1	84	2
324	2	180	2	1	1	1	84	3

325	3	0	1	1	1	1	48	2
326	1	280	2	1	1	2	80	3
327	1	0	1	1	1	1	73	3
328	1	240	2	1	1	3	35	2
329	3	0	2	1	1	2	68	2
330	2	240	1	1	1	4	60	2
331	3	0	2	1	1	1	77	3
332	3	0	3	1	3	4	55	2
333	1	260	2	1	1	4	28	1
334	1	120	1	1	1	1	80	2
335	3	0	2	1	1	3	53	3
336	1	260	2	1	1	3	28	1
337	1	260	2	1	2	3	28	1
338	1	260	2	1	1	3	28	1
339	3	0	1	1	1	1	24	2
340	2	244	2	1	1	2	51	2
341	2	280	1	1	1	1	48	3
342	3	0	2	1	1	1	74	3
343	3	0	2	1	2	1	72	3
344	3	280	2	1	1	1	36	1
345	3	0	1	1	1	1	58	2
346	3	0	2	1	1	1	58	3
347	3	0	2	1	1	1	79	3
348	3	0	2	1	1	4	36	2
349	1	280	2	1	1	1	80	3
350	2	240	2	1	1	1	73	2
351	1	240	2	1	1	2	72	3
352	3	0	2	1	1	1	79	3
353	3	200	1	1	1	2	72	3
354	1	280	2	1	2	4	62	2
355	3	0	2	1	1	1	65	3
356	1	260	2	1	2	4	62	1
357	3	200	2	1	1	4	71	3
358	2	0	1	1	1	1	77	2
359	3	180	2	1	1	1	82	2
360	3	280	1	1	1	1	48	2
361	3	240	2	1	1	1	80	2
362	2	240	2	1	1	1	67	2
363	3	0	2	1	3	4	62	2
364	3	0	1	1	1	1	68	2
365	3	240	1	1	1	1	79	2
366	3	270	1	1	1	1	77	3
367	3	0	1	1	1	1	81	2
368	3	0	2	1	2	3	79	2
369	3	260	2	1	1	1	69	2
370	3	0	1	1	1	1	72	2
371	3	0	2	1	1	1	52	2

372	2	260	3	1	1	1	48	2
373	2	260	2	1	1	1	83	3
374	3	0	2	1	1	3	76	3
375	3	0	2	1	1	2	79	2
376	3	0	2	1	1	1	81	2
377	3	0	2	1	1	1	84	3
378	3	0	2	1	1	3	67	3
379	3	0	1	1	1	1	66	3
380	3	160	2	1	1	1	76	3
381	1	0	2	1	1	1	77	3
382	3	270	2	1	1	3	42	2
383	3	240	2	1	1	1	76	3
384	3	275	2	1	1	1	41	2
385	3	280	2	1	1	1	70	2
386	1	0	1	1	1	1	67	2
387	3	0	2	1	1	1	59	3
388	3	260	2	1	1	3	55	2
389	3	240	2	1	1	1	56	3
390	3	0	2	1	1	1	72	3
391	3	0	2	1	1	2	60	2
392	3	0	1	1	1	1	79	3
393	2	140	2	1	2	4	84	3
394	3	0	1	1	1	1	82	3
395	3	0	1	1	1	1	78	3
396	3	0	1	1	1	1	72	2
397	3	240	2	1	1	1	68	3
398	3	0	2	1	1	1	59	3
399	3	230	2	1	1	1	55	2
400	3	0	1	1	1	3	72	3
401	3	260	1	1	1	1	67	3
402	1	240	2	1	1	1	74	3
403	3	120	2	1	1	1	71	2
404	3	240	1	1	1	4	36	2
405	3	0	2	1	1	1	79	3
406	3	0	2	1	1	1	69	2
407	3	0	1	1	1	1	66	3
408	1	240	2	1	1	1	74	3
409	3	0	2	1	2	2	60	2
410	3	0	1	1	1	1	66	3
411	3	0	2	1	1	3	80	2
412	3	0	2	1	1	1	67	2
413	3	0	2	1	1	2	77	3
414	3	200	2	1	1	1	71	3
415	3	0	1	1	1	1	82	3
416	3	260	1	1	1	1	74	2
417	3	0	2	1	1	1	48	1
418	3	224	2	1	1	1	74	3

419	3	0	2	1	1	2	62	2
420	3	260	2	1	1	1	70	3
421	3	196	1	1	1	1	12	2
422	3	200	1	1	1	1	48	3
423	3	240	2	1	1	1	59	3
424	3	40	2	1	2	2	47	2
425	3	0	2	1	1	1	83	3
426	3	280	2	1	2	4	61	2
427	3	240	2	1	1	3	31	2
428	3	0	2	1	1	1	80	3
429	3	100	2	1	1	1	71	3
430	1	260	2	1	1	1	84	3
431	2	160	2	1	1	4	64	2
432	3	260	2	1	1	1	74	3
433	3	0	2	1	3	4	57	2
434	2	240	2	1	3	4	61	2
435	3	0	1	1	1	4	60	2
436	3	0	2	1	2	4	51	2
437	3	160	2	1	1	2	77	3
438	3	80	2	1	2	4	63	3
439	3	0	2	1	1	4	68	2
440	3	0	1	1	1	2	80	2
441	3	0	1	1	1	2	77	3
442	1	268	2	2	3	4	65	2
443	3	240	2	1	3	4	56	2
444	3	0	3	1	2	4	82	3
445	3	0	1	1	3	2	74	2
446	1	263	2	1	3	4	70	2
447	3	0	2	1	3	4	68	2
448	3	0	2	1	1	2	75	3
449	3	0	2	1	2	4	52	2
450	3	0	2	1	1	2	60	3
451	3	230	2	1	2	4	60	2
452	2	240	1	1	2	3	69	3
453	3	0	2	1	1	4	62	2
454	3	0	2	1	1	3	73	2
455	3	280	2	1	2	4	56	2
456	3	96	2	1	1	2	56	3
457	3	0	2	1	1	4	59	2
458	3	120	2	1	1	3	84	3
459	3	20	2	1	2	4	60	2
460	3	0	2	1	3	4	52	2
461	3	0	2	1	2	4	77	3
462	3	0	2	1	3	4	63	2
463	3	0	1	1	1	3	69	3
464	3	0	2	1	2	4	69	3
465	3	0	2	1	1	2	76	3

466	3	0	2	1	1	2	65	2
467	3	80	2	1	2	4	65	2
468	3	0	2	1	2	4	63	3
469	3	0	2	1	1	2	73	3
470	3	0	2	2	1	4	71	3
471	3	0	2	1	2	4	70	3
472	3	260	2	1	1	4	84	3
473	3	200	2	1	3	4	43	2
474	3	0	2	1	2	4	66	3
475	3	0	2	1	1	4	59	2
476	3	180	2	1	2	4	78	3
477	3	0	2	1	1	2	76	3
478	3	270	2	1	1	4	76	3
479	3	0	2	1	2	4	82	3
480	3	0	1	1	1	2	80	2
481	1	268	2	1	3	4	65	2
482	3	280	2	1	2	4	56	2
483	3	260	2	1	1	4	84	3
484	3	80	2	1	2	4	62	3
485	3	0	2	1	1	2	76	3
486	3	0	2	1	2	4	52	2
487	3	0	2	1	2	4	63	3
488	3	260	2	1	1	4	84	3
489	3	80	2	1	2	4	63	3
490	3	20	2	1	2	4	60	2
491	2	240	1	1	2	3	69	3
492	2	240	1	1	2	3	69	3
493	3	0	2	1	1	2	73	3
494	3	0	1	1	1	2	84	3
495	3	120	2	1	1	2	79	3
496	3	0	2	2	2	4	78	2
497	3	0	2	1	2	4	81	3
498	3	0	2	1	1	4	84	3
499	3	260	2	2	3	4	59	2
500	3	0	2	1	1	3	76	3
501	3	80	2	1	2	4	24	1
502	3	0	2	1	2	4	48	2
503	3	0	2	2	1	4	48	2
504	3	0	2	1	1	2	84	3
505	3	0	2	1	2	4	79	3
506	3	0	2	1	3	4	54	2
507	3	0	1	1	1	2	64	2
508	3	260	2	1	2	4	80	3
509	3	0	1	1	1	2	48	3
510	3	0	2	1	3	4	64	2
511	3	0	2	1	3	4	78	3
512	3	240	2	1	2	4	75	3

513	3	0	2	1	1	2	73	3
514	3	160	2	1	3	4	64	2
515	3	0	2	1	3	4	60	2
516	3	0	2	1	2	4	29	2
517	3	0	2	1	3	4	84	2
518	3	240	2	1	2	4	73	3
519	3	0	2	1	2	4	83	3
520	3	280	2	1	2	4	64	2
521	3	0	1	1	2	4	48	1
522	3	0	2	1	2	4	46	2
523	3	0	2	1	1	4	59	2
524	3	0	1	1	1	2	69	2
525	3	0	1	1	1	2	69	2
526	3	0	2	1	2	4	12	1
527	3	0	2	1	1	4	81	2
528	3	0	2	1	1	4	70	2
529	3	0	2	1	3	4	59	2
530	3	0	2	1	1	4	80	3
531	3	0	2	1	2	4	62	2
532	3	0	2	1	2	4	60	2
533	3	0	2	1	1	4	74	2
534	3	0	2	1	3	4	48	2
535	3	64	2	1	2	4	72	2
536	3	0	1	1	1	2	75	3
537	3	140	1	1	1	2	84	3
538	3	240	1	1	1	4	48	2
539	3	0	2	1	1	2	84	3
540	3	0	2	2	3	4	62	2
541	3	240	2	1	2	4	76	2
542	2	280	2	1	2	4	80	3
543	3	0	2	1	3	4	69	2
544	3	0	2	1	2	4	74	2
545	3	0	1	1	2	4	40	2
546	3	160	2	1	2	4	72	2
547	3	0	2	1	3	4	78	3
548	3	240	1	1	1	4	48	2
549	3	0	2	1	2	4	46	2
550	3	0	2	1	2	4	59	2
551	3	0	2	1	3	4	60	2
552	3	260	2	2	3	4	49	2
553	3	240	2	1	2	4	75	3
554	3	0	2	1	1	2	67	3
555	3	240	2	1	1	3	81	3
556	1	248	2	1	2	4	67	3
557	3	270	2	1	2	4	81	3
558	3	0	1	1	1	4	84	1
559	1	240	2	1	2	2	77	3

560	3	248	2	1	2	4	84	3
561	1	0	2	1	2	4	77	2
562	3	0	1	1	1	4	84	1
563	3	0	2	1	1	3	72	3
564	3	0	2	2	3	4	70	2
565	3	0	1	1	1	4	84	1
566	3	140	2	1	2	4	80	3
567	3	120	2	1	2	4	64	3
568	3	0	2	1	1	2	82	3
569	3	0	2	1	2	4	72	2
570	3	0	1	1	3	4	49	2
571	3	240	1	1	1	2	84	3
572	3	240	2	1	1	2	82	3
573	2	240	1	1	1	2	66	3
574	3	240	2	1	2	4	77	2
575	3	270	2	1	2	4	78	3
576	3	60	2	1	1	4	57	3
577	3	0	2	1	2	4	72	2
578	3	280	2	1	1	3	70	3
579	3	275	2	1	3	4	73	3
580	2	0	2	1	3	4	80	2
581	3	160	2	1	2	4	47	2
582	3	80	2	1	2	4	57	2
583	3	80	1	1	2	2	82	3
584	3	180	1	1	1	4	84	3
585	3	0	2	1	2	4	84	3
586	3	0	2	1	2	4	72	2
587	2	0	2	1	1	2	69	3
588	3	260	2	1	2	4	72	3
589	3	0	2	1	3	4	69	2
590	3	240	2	1	2	4	59	3
591	3	270	2	1	1	3	70	3
592	3	0	2	1	1	4	67	2
593	3	0	2	1	2	4	64	2
594	3	196	1	1	1	2	48	2
595	3	0	2	1	1	4	62	2
596	2	240	2	1	1	4	68	2
597	2	120	2	1	2	4	69	3
598	3	0	1	1	1	4	84	3
599	3	0	2	1	2	4	76	2
600	3	0	1	1	1	4	80	3
601	3	0	2	1	2	4	60	3
602	3	40	2	1	2	4	53	2
603	3	120	2	1	2	4	37	2
604	3	0	3	1	1	3	84	3
605	3	80	2	1	2	4	32	3
606	3	0	2	1	2	3	77	2

607	3	280	2	1	2	4	56	2
608	3	260	2	1	2	4	56	2
609	3	0	2	1	1	4	59	2
610	3	0	2	1	1	1	72	2
611	3	0	1	1	1	1	60	3
612	3	0	1	1	1	1	60	3
613	3	0	2	1	1	1	70	2
614	3	0	2	1	1	4	75	2
615	3	260	2	1	1	1	80	3
616	3	0	2	1	1	1	64	3
617	1	240	1	1	1	1	72	3
618	3	0	2	1	1	1	63	3
619	3	0	1	1	1	1	84	3
620	2	0	1	1	2	4	75	2
621	3	280	2	1	1	1	53	2
622	3	0	2	1	2	4	26	2
623	3	200	1	1	1	1	75	3
624	1	270	2	1	1	1	79	2
625	1	248	1	1	1	2	36	1
626	1	210	1	1	1	1	36	2
627	3	268	2	1	2	4	74	2
628	3	0	1	1	1	1	72	3
629	3	240	1	1	1	1	84	3
630	1	250	2	1	1	1	24	1
631	3	0	2	1	1	3	80	2
632	3	0	2	1	1	1	71	3
633	3	270	3	2	1	4	25	2
634	3	60	2	1	1	1	73	2
635	2	0	2	1	1	1	84	3
636	2	0	2	1	1	1	70	3
637	3	80	1	1	1	1	79	3
638	3	0	2	2	3	4	30	2
639	3	0	1	1	1	1	56	3
640	3	140	2	1	1	3	18	3
641	3	140	2	1	1	3	18	3
642	3	80	2	1	1	4	64	2
643	3	0	2	1	1	1	72	3
644	3	0	2	1	1	1	62	3
645	3	270	2	1	1	1	73	2
646	3	278	2	1	1	1	73	3
647	3	260	2	1	1	3	63	2
648	3	240	2	1	1	1	73	3
649	3	260	2	1	1	4	78	2
650	3	0	2	1	1	1	72	2
651	3	268	1	1	1	1	68	2
652	3	0	2	1	1	1	71	3
653	1	264	1	1	1	2	72	2

654	3	120	1	1	1	1	66	2
655	3	240	2	1	1	1	72	3
656	2	240	1	1	1	1	78	3
657	3	0	2	1	1	1	67	3
658	3	0	2	1	1	4	75	3
659	3	280	2	1	1	1	75	2
660	3	0	2	1	1	1	72	2
661	3	240	2	1	1	1	74	2
662	3	0	2	1	1	4	72	2
663	3	240	2	1	1	2	45	2
664	3	280	2	1	1	1	75	2
665	3	36	3	1	3	4	60	2
666	2	248	2	1	1	2	84	2
667	3	230	2	1	1	1	82	3
668	3	120	2	1	2	4	32	2
669	3	120	2	1	1	2	32	3
670	3	120	2	1	1	2	75	3
671	2	0	2	1	1	1	70	3
672	3	24	2	1	1	4	58	3
673	2	0	1	1	1	1	36	2
674	1	280	2	2	2	4	63	2
675	3	80	2	1	1	2	74	3
676	3	80	2	1	1	2	48	1
677	3	0	2	1	1	1	83	2
678	3	0	2	1	1	2	55	2
679	3	0	2	1	1	3	72	2
680	3	0	1	1	2	4	72	1
681	1	200	1	1	1	2	72	2
682	3	0	2	1	1	1	73	3
683	3	0	2	1	1	2	68	2
684	3	0	1	1	1	1	80	3
685	2	80	2	1	1	1	82	3
686	3	0	2	1	1	1	83	2
687	3	260	2	1	1	1	73	3
688	3	0	2	1	1	3	48	1
689	3	120	2	1	1	1	77	2
690	3	0	2	1	1	1	72	2
691	3	80	2	1	1	3	48	1
692	1	263	2	1	1	1	48	2
693	2	0	2	1	1	1	63	3
694	1	275	1	1	1	2	84	2
695	3	0	1	1	1	1	48	2
696	2	280	1	1	1	1	59	3
697	3	0	1	1	1	1	78	1
698	2	0	2	1	1	1	75	2
699	2	120	1	1	1	1	76	3
700	2	230	2	1	1	1	80	3

701	2	260	2	1	1	2	75	3
702	2	0	2	1	1	4	24	2
703	1	240	2	1	1	1	65	3
704	3	240	1	1	1	1	78	3
705	1	280	2	1	1	2	60	2
706	2	120	1	1	1	1	76	3
707	1	260	2	1	1	4	76	2
708	3	0	1	1	1	3	73	2
709	1	220	1	1	1	4	51	2
710	3	0	3	2	3	4	60	3
711	3	120	3	1	1	1	60	3
712	3	0	2	2	1	4	26	1
713	3	0	2	1	1	2	61	2
714	3	0	2	1	1	2	61	2
715	3	0	1	1	1	3	48	2
716	2	0	2	1	1	1	70	3
717	2	220	2	1	1	1	71	3
718	3	0	2	1	1	2	48	2
719	3	0	2	1	1	1	80	3
720	1	260	1	1	1	1	72	3
721	3	0	2	1	1	3	72	2
722	3	280	2	1	1	2	48	2
723	3	0	2	1	1	1	62	2
724	3	0	2	1	1	1	72	3
725	2	160	2	1	1	3	52	2
726	3	0	2	2	1	1	79	3
727	3	280	2	1	1	1	60	2
728	3	0	2	1	2	4	48	2
729	3	280	2	1	1	1	60	2
730	3	240	1	1	1	2	72	3
731	3	260	2	1	2	4	53	2
732	2	240	2	1	1	1	62	2
733	3	260	2	1	2	4	54	2
734	1	0	1	1	1	2	84	3
735	3	120	1	1	1	1	78	3
736	3	240	3	1	2	4	48	2
737	3	0	2	1	1	1	48	2
738	3	0	2	1	1	1	72	3
739	3	0	2	1	1	1	77	3
740	2	0	2	1	1	1	78	3
741	2	260	2	1	1	2	72	2
742	3	220	2	1	1	4	69	2
743	3	220	2	1	1	2	62	2
744	2	0	2	1	1	1	73	3
745	3	0	2	1	1	1	69	2
746	3	0	2	1	1	4	52	2
747	3	260	2	1	1	2	77	3

748	3	0	1	1	1	1	78	3
749	2	240	2	1	1	4	72	2
750	3	0	2	1	1	4	71	2
751	3	232	2	1	3	4	73	2
752	3	0	2	1	1	2	70	2
753	3	0	2	1	1	2	48	1
754	3	0	2	1	1	1	60	3
755	3	0	3	2	2	4	81	2
756	2	240	1	1	1	1	36	2
757	3	230	1	1	1	1	83	3
758	3	230	2	1	1	4	56	3
759	2	240	2	1	1	1	71	3
760	1	280	2	1	1	1	71	3
761	1	0	2	1	1	1	76	2
762	3	240	2	1	1	3	60	2
763	2	230	2	1	1	2	73	3
764	1	240	2	1	1	1	77	3
765	2	240	2	1	1	2	70	3
766	3	0	2	1	1	2	77	3
767	3	120	2	1	1	2	71	2
768	3	0	2	1	1	1	48	3
769	3	0	2	1	1	1	61	2
770	3	260	2	1	1	1	81	3
771	3	260	2	1	1	1	69	3
772	2	260	2	1	1	2	60	2
773	2	0	2	1	1	1	74	3
774	3	248	1	1	1	1	84	3
775	2	220	2	1	1	1	76	3
776	3	0	2	1	1	2	61	3
777	1	0	2	1	1	3	57	2
778	3	240	2	1	1	1	77	3
779	3	0	2	1	2	4	75	2
780	2	230	1	1	1	1	84	3
781	2	230	1	1	1	1	77	3
782	3	0	2	1	1	2	58	2
783	3	0	1	1	1	2	71	2
784	3	0	1	1	1	1	71	2
785	3	0	2	1	1	1	68	3
786	3	0	2	1	1	1	66	3
787	3	120	2	1	1	1	36	1
788	3	280	3	1	1	3	34	2
789	3	270	1	1	1	1	82	3
790	3	280	2	1	1	1	59	3
791	1	264	2	1	1	1	84	3
792	2	280	2	1	1	1	77	3
793	3	0	2	1	1	4	65	2
794	3	0	2	1	1	1	64	2

795	3	0	3	1	1	4	36	3
796	3	0	2	1	3	4	47	2
797	3	0	2	1	1	1	61	3
798	2	0	2	1	1	2	47	2
799	2	220	2	1	1	3	70	2
800	3	0	2	1	1	3	84	3
801	3	64	2	1	1	1	71	3
802	1	240	2	1	1	1	47	2
803	3	260	1	1	1	1	82	3
804	3	280	2	1	1	2	65	2
805	3	0	2	1	1	3	65	2
806	3	0	2	1	1	3	78	2
807	3	80	2	1	1	4	57	2
808	3	0	1	1	1	1	59	3
809	3	240	2	1	1	1	84	3
810	3	280	2	1	1	3	67	2
811	3	0	2	1	1	1	84	3
812	2	0	2	1	1	4	57	2
813	1	270	2	1	1	1	84	3
814	1	80	2	1	1	1	60	3
815	3	0	2	1	1	2	64	3
816	1	260	2	1	1	1	72	2
817	2	240	2	1	3	4	42	2
818	3	0	2	1	1	1	75	2
819	3	0	2	1	1	4	48	2
820	3	12	1	1	1	1	80	3
821	2	240	2	1	1	3	75	3
822	1	280	3	1	2	4	62	2
823	3	0	2	1	1	4	53	2
824	3	220	2	1	2	4	66	2
825	2	270	2	1	1	2	63	3
826	3	0	2	1	1	1	74	3
827	3	0	2	1	1	1	80	2
828	2	260	2	1	1	2	79	3
829	3	0	2	1	1	2	59	2
830	3	0	2	2	1	4	36	2
831	1	0	2	1	1	1	54	3
832	3	240	2	1	1	1	80	2
833	3	260	2	2	3	4	34	2
834	1	0	2	1	1	2	74	3
835	3	270	2	1	2	4	51	3
836	2	200	2	1	1	1	68	3
837	3	280	2	1	1	4	80	2
838	2	0	2	1	1	2	61	2
839	3	0	1	2	1	4	84	2
840	3	0	2	1	1	1	72	2
841	3	0	2	1	1	2	84	3

842	1	0	2	1	1	1	54	3
843	3	280	2	2	1	3	60	2
844	3	0	2	1	1	2	53	2
845	3	280	2	1	1	2	76	2
846	3	0	3	1	3	4	51	2
847	3	120	2	1	2	4	50	2
848	3	0	2	1	1	1	48	3
849	3	0	2	1	1	1	12	1
850	3	260	1	1	1	1	72	3
851	3	0	2	1	1	2	48	2
852	2	260	2	1	1	2	73	2
853	2	0	2	1	1	2	72	3
854	3	0	2	1	1	1	77	3
855	3	0	2	1	2	4	57	2
856	1	80	2	1	1	1	60	3
857	3	0	2	1	1	4	79	3
858	3	0	3	1	1	3	76	2
859	3	0	1	1	1	1	36	1
860	3	0	2	1	1	3	72	2
861	3	240	2	1	1	1	83	3
862	3	240	2	1	1	1	38	3
863	3	0	2	1	1	4	72	2
864	3	0	3	1	3	4	72	2
865	3	240	2	1	1	3	72	2
866	3	0	2	1	1	4	84	2
867	3	0	1	1	1	2	70	2
868	3	0	1	1	1	1	76	3
869	3	0	2	1	1	4	57	2
870	3	0	1	1	1	1	53	2
871	3	0	2	1	1	1	48	2
872	2	0	2	1	1	2	72	3
873	3	240	2	1	2	4	72	2
874	3	0	2	2	2	3	73	3
875	3	0	2	1	1	1	60	3
876	2	240	2	1	1	1	36	2
877	3	240	2	1	1	1	84	3
878	3	240	2	1	1	4	68	2
879	1	280	2	1	1	1	77	3
880	3	120	1	1	1	2	78	3
881	3	0	2	1	1	1	59	2
882	1	240	2	1	1	1	81	3
883	3	0	2	1	1	3	62	2
884	2	0	1	1	1	2	71	1
885	3	100	2	1	2	4	28	2
886	2	280	2	2	1	4	36	1
887	2	220	1	1	2	1	42	2
888	3	0	2	1	1	1	67	2

889	3	200	2	1	1	1	79	3
890	3	240	2	1	1	1	50	2
891	3	0	2	1	1	1	81	2
892	3	168	3	1	2	4	69	2
893	3	0	2	1	1	1	78	3
894	3	280	2	1	1	2	73	2
895	3	0	1	1	1	1	77	2
896	3	180	2	1	2	4	84	2
897	3	0	2	1	2	4	57	2
898	2	260	2	1	1	1	83	3
899	2	0	2	1	1	1	74	3
900	3	0	2	1	1	1	73	2
901	3	240	2	1	1	1	72	3
902	3	0	2	1	1	1	81	3
903	2	120	1	1	1	1	47	1
904	2	240	2	1	1	3	75	3
905	3	0	2	2	2	4	84	2
906	3	0	2	1	1	3	72	2
907	1	240	1	1	1	1	72	2
908	3	0	2	1	1	3	60	2
909	3	0	1	1	1	1	75	2
910	2	244	2	1	1	1	70	3
911	2	270	1	1	1	1	65	2
912	3	280	1	1	1	1	74	3
913	2	240	1	1	1	1	77	3
914	3	280	1	1	1	1	63	3
915	3	0	1	1	1	2	80	2
916	3	230	1	1	1	1	79	3
917	2	240	1	1	1	1	53	2
918	2	250	1	1	1	1	73	3
919	3	0	1	1	1	2	60	3
920	3	0	1	1	1	2	72	3
921	3	230	1	1	1	1	73	3
922	3	0	1	1	1	1	67	3
923	3	0	1	1	1	1	77	3
924	1	250	1	1	1	1	77	3
925	2	200	2	1	1	2	67	2
926	3	0	1	1	1	1	76	3
927	3	0	1	1	1	1	72	2
928	3	0	2	1	1	2	79	3
929	2	200	1	1	1	1	73	3
930	3	80	1	1	1	2	77	3
931	3	0	2	1	1	2	74	3
932	3	80	1	1	1	1	78	3
933	3	240	1	1	1	1	60	2
934	3	0	1	1	1	2	84	3
935	3	0	2	1	1	1	56	3

936	3	0	1	1	1	2	56	3
937	3	0	1	1	1	2	65	3
938	3	0	2	1	1	2	67	3
939	3	0	1	1	1	1	72	2
940	3	80	1	1	1	1	73	3
941	3	240	1	1	1	2	68	3
942	3	120	1	1	1	2	56	2
943	1	280	2	1	1	4	66	2
944	1	220	2	1	1	1	67	2
945	2	160	1	1	1	2	74	3
946	3	0	1	1	1	2	74	2
947	1	200	1	1	1	2	59	3
948	3	0	1	1	1	2	65	2
949	2	240	2	1	1	1	72	2
950	3	0	1	1	1	1	60	3
951	3	0	1	1	1	1	70	3
952	3	280	2	1	1	1	74	3
953	3	0	1	1	1	2	60	3
954	3	230	1	1	1	3	56	3
955	2	240	1	1	1	2	71	3
956	3	240	1	1	1	1	63	3
957	3	160	2	1	1	1	73	2
958	3	0	2	1	1	2	66	3
959	2	280	1	1	1	1	69	3
960	2	60	2	1	1	1	60	2
961	2	80	2	1	1	1	73	2
962	3	280	1	1	1	2	49	2
963	3	0	1	1	1	1	84	3
964	2	280	1	1	1	1	69	3
965	3	0	2	1	1	2	74	3
966	2	0	2	1	1	1	68	3
967	2	120	1	1	1	2	48	2
968	3	0	1	1	1	2	52	3
969	3	0	1	1	1	2	51	2
970	3	200	2	1	1	2	68	2
971	3	180	1	1	1	1	65	2
972	1	280	2	1	1	1	69	2
973	3	240	2	1	1	2	66	2
974	3	160	1	1	1	2	61	3
975	3	240	2	1	1	2	66	2
976	3	260	2	1	1	3	62	2
977	3	280	1	1	1	2	79	3
978	3	0	1	1	1	1	84	3
979	3	60	2	1	1	3	62	2
980	3	268	1	1	1	1	60	2
981	3	0	1	1	1	2	51	2
982	1	0	2	1	2	2	46	2

983	3	140	1	1	1	1	19	1
984	1	0	1	1	1	2	72	3
985	1	280	2	1	1	2	48	2
986	1	240	2	1	1	1	38	2
987	3	0	2	2	1	2	29	2
988	3	0	1	1	1	2	51	2
989	3	32	2	1	1	3	72	2
990	3	0	2	1	1	2	79	3
991	2	260	1	1	1	2	35	2
992	3	0	3	2	3	4	84	2
993	3	0	2	1	1	1	77	3
994	3	0	2	1	1	1	77	3
995	3	0	1	1	1	3	40	2
996	3	240	2	1	1	3	67	3
997	2	260	3	2	3	4	63	2
998	3	20	2	1	1	3	72	3
999	3	0	2	1	1	1	60	2
1000	3	180	2	1	1	1	59	2
1001	3	180	2	1	1	2	60	2

IPAQ-SF: 1 is "sedentary", 2 is "moderate", and 3 is "high"; PSS-10: 1 is "low", 2 is "moderate", and 3 is "high"; MDQ: 1 is "normal" and 2 is "bipolar"; GAD-7: 1 is "normal", 2 is "moderate", and 3 is "high"; PHQ-9: 1 is "no symptoms", 2 is "mild", 3 is "moderate", and 4 is "severe"; WHOQOL-BREF: 1 is "poor", 2 is "moderate", and 3 is "good"
