# Supplementary material for: Awareness of COVID-19 influences on the wellness of Thai health professional students: An ambulatory assessment during the early “new normal” informing policy
Source: PLoS One. 2021 Jun 14;16(6):e0252681. doi: 10.1371/journal.pone.0252681 (PMC8202936; doi:10.1371/journal.pone.0252681)
Supplement: S4 Raw data — (PDF) [file pone.0252681.s006.pdf]

S4 Raw data. Association between COVID-19 awareness and physical activity, stress, mood, symptoms of anxiety and depression, social well-being, and quality of life: Stress, mood, anxiety symptoms, depression symptoms, and quality of life_T5 Attitude towards the COVID-19 pandemic and its influence on anxiety, social well-being, and quality of life among Thai health professional students during the early “new normal” informing policy: GPAX, income, anxiety symptoms, and quality of life_S2 Knowledge of COVID-19 influences on social well-being and quality of life among the Thai health professional students during the early “new normal” informing policy: GPAX, income, and quality of life

No	GPAX	Income	Stress	Mood	Anxiety	Depresion	Quality of life
1	3.26	6000	16	7	5	7	89
2	2.98	5000	18	0	6	27	91
3	3.32	16000	7	1	0	1	98
4	3.37	10000	10	0	1	2	102
5	3.53	6000	20	6	3	9	73
6	3.09	3000	22	4	14	14	89
7	3.01	5000	14	10	0	4	82
8	2.90	4000	0	0	0	3	41
9	3.30	3000	17	2	0	0	118
10	3.02	5000	26	5	4	3	105
11	3.28	3000	26	13	13	25	76
12	2.94	6000	20	1	14	18	78
13	3.31	5000	20	0	0	0	104
14	3.03	6000	20	8	7	10	78
15	3.13	8000	27	0	7	18	89
16	3.00	7000	11	4	0	1	88
17	3.25	6000	20	4	0	0	72
18	3.04	6000	14	2	0	3	79
19	3.22	5000	18	3	2	22	75
20	3.00	5000	10	5	0	9	97
21	2.90	10000	22	2	3	2	123
22	3.30	5000	27	7	7	14	78
23	3.18	6000	19	16	6	17	95
24	2.82	3000	21	1	0	11	64
25	3.00	3000	16	5	2	6	56
26	3.17	4000	16	8	0	0	96
27	2.83	4000	21	4	4	16	92
28	3.29	4000	21	0	0	11	78
29	3.36	6000	19	9	0	1	121
30	3.39	6000	20	1	7	14	58
31	2.81	3000	29	8	7	27	99
32	2.90	10000	28	2	9	27	79
33	2.90	3000	11	1	1	4	100
34	3.30	3000	12	4	0	0	40
35	3.04	6000	20	1	2	11	112
36	3.08	6000	24	3	7	15	93
37	3.29	4000	14	1	2	0	100
38	3.14	3000	3	5	0	0	78

39	3.29	4000	16	0	0	1	111
40	3.01	5000	13	7	0	0	85
41	2.90	4000	23	0	4	17	91
42	2.90	10000	17	4	1	14	92
43	2.90	4000	18	6	0	0	113
44	3.41	10000	10	6	2	5	98
45	2.90	3000	12	0	7	18	77
46	3.13	8000	16	5	0	3	101
47	3.03	6000	28	4	21	0	78
48	3.08	6000	16	3	0	1	78
49	3.28	6000	19	11	4	24	104
50	3.00	8000	24	9	7	19	78
51	2.55	8000	0	11	0	8	124
52	3.11	3000	13	6	9	23	64
53	2.97	3000	23	8	14	14	81
54	2.64	6000	22	4	8	27	71
55	3.21	4000	20	13	7	18	86
56	2.88	4500	22	6	7	18	87
57	2.67	3000	15	3	0	0	78
58	2.98	7000	22	4	0	4	84
59	2.95	3500	24	14	2	8	92
60	2.61	3000	14	0	0	2	76
61	3.19	3000	15	5	0	0	79
62	3.02	3500	9	8	2	8	85
63	2.63	4500	18	7	2	6	95
64	2.77	6000	24	3	16	15	82
65	3.01	3500	16	6	3	4	99
66	3.65	4500	24	1	3	8	85
67	2.82	6000	18	7	0	0	116
68	3.02	5000	24	3	5	18	88
69	2.55	4000	14	6	1	7	100
70	2.63	6000	16	8	8	8	74
71	3.11	6000	25	0	7	18	74
72	2.86	4000	16	9	3	3	95
73	2.55	8000	20	4	9	27	82
74	2.54	4500	17	2	8	15	89
75	2.66	4000	20	0	5	21	85
76	2.76	4000	16	4	5	7	91
77	2.76	4000	18	4	0	0	113
78	3.20	4000	16	2	0	1	78
79	3.08	6000	27	3	7	14	78
80	2.47	11000	14	4	1	0	100
81	3.94	3000	20	8	0	0	72
82	3.84	8000	28	1	9	27	79
83	3.80	9000	16	3	2	6	56
84	2.30	7000	16	0	3	4	99
85	3.77	12000	7	3	0	1	98

86	3.86	4000	16	8	3	4	99
87	3.70	4000	27	0	7	18	90
88	3.75	10000	11	4	0	1	88
89	3.65	12000	18	7	0	0	113
90	3.84	4000	23	0	4	17	91
91	3.66	6430	11	6	4	1	88
92	3.79	3000	22	4	14	14	89
93	2.21	8000	16	5	0	1	78
94	3.54	5000	16	4	0	0	96
95	3.74	2500	21	8	0	11	78
96	3.81	4000	21	5	4	16	92
97	3.93	6000	26	7	13	25	74
98	3.84	6000	18	1	6	27	91
99	3.86	8000	16	1	1	6	56
100	3.82	6000	16	6	1	6	56
101	3.68	5000	15	0	0	0	78
102	3.81	6000	17	0	1	14	92
103	3.92	8000	17	14	1	14	91
104	3.54	6000	10	16	0	0	78
105	3.62	5000	19	2	7	7	64
106	2.31	3000	20	0	3	10	109
107	3.93	5000	2	4	0	0	121
108	3.60	3000	18	0	10	17	80
109	3.93	5000	20	4	7	18	78
110	3.87	4500	13	17	6	13	99
111	3.73	8000	11	3	7	16	78
112	3.92	4500	19	7	10	23	93
113	3.66	9000	22	9	11	18	83
114	3.92	6000	20	1	8	20	84
115	3.87	6000	17	9	1	7	101
116	3.60	4000	24	5	8	11	83
117	3.60	4000	15	1	8	20	74
118	3.68	5200	21	2	4	17	78
119	3.67	3000	16	1	6	9	78
120	3.77	6000	14	1	0	0	102
121	3.98	8000	13	1	4	7	91
122	3.60	5000	13	2	4	7	86
123	3.58	2000	22	2	5	12	94
124	3.71	6000	17	3	3	4	71
125	3.61	5000	22	2	3	15	86
126	3.63	5000	16	1	2	3	96
127	3.73	6000	5	6	0	3	93
128	3.73	5000	26	4	7	14	62
129	3.73	6000	13	10	6	13	99
130	3.90	4000	16	4	6	17	96
131	3.78	6000	18	6	10	17	80
132	3.69	4500	18	0	6	5	94

133	3.97	4000	29	14	1	0	103
134	3.72	7000	10	1	0	0	78
135	3.72	5000	5	6	0	3	94
136	3.81	6000	27	6	7	14	64
137	3.90	4000	25	0	1	2	101
138	3.66	5000	21	0	3	6	107
139	3.67	4000	21	1	3	6	107
140	3.69	6000	5	0	0	3	93
141	3.74	3000	19	5	3	6	107
142	3.82	4000	14	8	3	0	76
143	3.57	5000	20	3	4	5	86
144	3.64	4000	19	5	0	7	63
145	3.65	6000	14	0	0	0	106
146	3.49	5000	17	5	1	3	78
147	3.74	6000	19	5	2	10	98
148	3.09	3000	18	14	1	6	115
149	3.83	3000	32	6	0	20	34
150	3.28	6500	15	5	5	6	67
151	3.64	7000	21	5	3	9	85
152	3.52	6000	20	6	9	11	80
153	3.60	8000	20	9	11	19	65
154	3.52	6000	26	5	7	15	76
155	3.49	5000	16	5	7	26	123
156	3.64	4000	23	1	0	1	113
157	3.40	9000	20	6	4	10	106
158	3.03	6000	17	3	0	5	105
159	3.62	12000	13	0	1	8	88
160	3.11	10000	17	0	2	0	88
161	3.11	10000	17	4	1	0	122
162	3.45	5000	0	0	0	2	123
163	3.21	6000	14	5	1	0	99
164	3.80	9000	20	0	7	9	78
165	3.74	5000	15	6	2	0	82
166	3.39	7000	8	7	4	6	96
167	3.31	8000	22	0	6	8	101
168	3.31	7000	7	8	0	1	97
169	3.31	7000	25	1	21	27	83
170	3.42	2500	19	3	0	13	104
171	3.63	3000	16	3	1	1	102
172	2.92	5000	13	4	3	3	107
173	3.40	5000	0	4	0	1	105
174	3.32	8000	23	9	0	0	113
175	3.72	6000	23	0	7	10	91
176	3.12	6000	16	0	1	5	100
177	3.61	5000	20	4	7	9	93
178	3.12	6000	16	8	2	2	78
179	2.90	3000	23	6	9	16	86

180	3.59	4000	17	4	6	6	89
181	3.49	6000	9	6	2	1	91
182	3.50	5000	14	10	0	0	119
183	3.76	4000	23	1	4	24	76
184	3.77	8000	13	8	1	0	108
185	3.15	9000	16	2	4	6	91
186	2.88	5000	20	0	0	4	82
187	3.83	3000	5	1	0	0	107
188	3.25	2300	17	3	1	0	113
189	3.42	1500	16	11	0	1	98
190	3.02	6000	23	3	13	26	72
191	3.33	5000	18	8	13	7	103
192	3.56	3600	16	9	5	13	107
193	3.21	4000	15	1	12	18	72
194	3.51	6000	28	0	0	0	78
195	3.26	5000	18	1	0	2	120
196	3.81	6000	20	0	7	18	80
197	3.71	3000	16	5	0	0	96
198	3.71	9000	15	5	3	10	75
199	3.62	3500	20	1	6	12	89
200	3.47	3500	17	0	0	0	118
201	3.41	4000	10	3	0	0	88
202	3.37	6000	19	9	6	4	106
203	3.83	10000	16	6	12	25	107
204	3.48	4000	21	6	5	12	78
205	3.52	4000	12	5	0	0	100
206	3.20	7000	8	0	0	0	106
207	3.10	4000	19	1	2	1	107
208	3.83	3000	22	2	1	3	106
209	3.29	5000	22	5	6	2	92
210	3.30	3000	16	0	5	8	57
211	3.25	3000	12	2	0	0	118
212	3.43	4000	16	8	0	2	83
213	3.78	3000	22	4	9	27	75
214	2.24	5000	10	4	7	9	74
215	3.03	5000	8	1	0	0	104
216	2.66	5000	19	12	2	5	87
217	2.96	5000	26	16	8	14	85
218	3.67	2500	20	7	12	27	82
219	2.98	50000	22	4	7	21	88
220	3.58	5000	16	1	1	0	76
221	3.77	3500	17	5	0	0	118
222	3.11	5000	16	6	4	8	88
223	2.80	3000	30	2	10	9	113
224	2.94	4000	14	2	3	4	81
225	3.16	5000	24	9	2	5	89
226	2.97	7000	21	0	9	17	85

227	3.01	6000	15	0	0	2	96
228	2.85	6000	22	12	3	0	85
229	2.65	4000	17	4	9	12	79
230	2.70	5000	19	6	8	9	89
231	2.70	6000	10	1	0	1	89
232	2.59	6000	17	0	4	4	108
233	3.25	8000	10	9	0	0	99
234	2.76	10000	23	0	6	11	91
235	3.44	6000	15	1	2	5	90
236	3.07	5000	21	6	0	5	99
237	3.03	6000	23	0	9	20	87
238	3.03	3600	14	3	1	2	99
239	4.00	6000	19	5	4	2	89
240	3.38	5000	15	2	0	3	81
241	2.88	6000	18	0	0	4	89
242	2.66	5000	16	10	0	0	107
243	3.33	5000	23	6	6	23	101
244	2.42	2400	18	4	7	3	79
245	3.44	6000	17	4	4	15	82
246	2.66	7000	16	2	2	5	94
247	2.14	4000	17	8	3	8	83
248	2.66	8000	17	8	6	5	95
249	2.74	5000	19	0	18	20	95
250	2.14	4000	10	5	0	0	85
251	3.14	4800	21	2	1	0	97
252	3.46	6000	18	4	7	19	77
253	3.18	4500	17	9	8	16	73
254	2.70	6000	19	2	6	10	87
255	2.65	4000	12	13	2	3	79
256	3.03	6000	11	5	4	9	45
257	3.16	5000	17	2	8	10	65
258	2.66	8000	12	12	0	2	90
259	3.16	5000	15	10	7	3	56
260	3.07	5000	22	2	4	19	100
261	2.62	9000	20	12	4	2	122
262	2.93	10000	17	0	9	12	77
263	3.00	4000	15	4	3	0	113
264	2.04	10000	19	3	2	2	100
265	3.00	4000	19	0	1	7	57
266	2.38	7500	18	8	0	4	103
267	2.73	6000	17	0	6	5	96
268	2.50	6000	28	2	0	0	78
269	3.03	4000	15	6	0	1	86
270	2.75	10000	23	7	9	20	87
271	3.32	6000	13	9	3	10	90
272	2.50	5000	21	8	9	17	85
273	2.50	3000	10	8	7	14	55

274	2.50	3000	26	8	9	27	101
275	2.34	10000	17	0	7	7	78
276	3.07	8000	11	0	1	4	88
277	3.56	5000	20	12	1	0	110
278	2.80	4000	21	1	7	22	104
279	2.28	5000	14	11	0	0	100
280	2.40	6000	23	0	13	26	72
281	2.56	3000	20	11	0	0	118
282	3.60	5000	31	1	17	27	92
283	2.58	5000	18	1	2	0	106
284	2.54	5000	24	6	0	1	116
285	3.25	5000	21	2	9	12	92
286	3.18	4500	13	1	2	3	92
287	2.38	7500	20	15	0	0	78
288	3.32	6000	10	0	7	9	89
289	2.28	5000	11	7	0	0	103
290	3.38	6000	17	4	9	11	89
291	2.98	4000	14	7	9	3	112
292	2.73	6000	25	7	6	19	93
293	2.50	6000	20	5	8	12	104
294	3.10	4000	16	1	0	0	122
295	2.66	4000	10	9	0	0	123
296	2.93	6000	15	1	1	0	98
297	2.68	9000	20	4	0	2	102
298	3.69	6000	11	3	0	0	97
299	3.07	6000	20	6	7	9	98
300	2.29	10000	30	0	0	0	90
301	3.00	10000	20	13	7	16	79
302	2.00	9000	14	0	9	17	78
303	2.70	8000	18	11	0	2	113
304	3.31	8000	31	8	17	27	93
305	3.55	6500	16	11	3	5	100
306	2.88	12000	31	7	17	27	93
307	3.02	4000	25	0	6	20	93
308	3.39	6000	11	0	0	0	103
309	2.77	8000	11	4	0	0	103
310	3.45	4000	17	8	2	4	82
311	3.67	3500	6	2	0	0	80
312	3.07	4000	11	7	0	0	78
313	3.37	31000	40	3	11	23	65
314	3.23	6000	24	3	10	10	91
315	3.30	10000	17	3	0	0	106
316	2.27	6500	24	15	12	10	91
317	3.42	3000	3	4	0	0	98
318	3.82	5000	22	3	7	9	76
319	3.30	5000	24	6	12	10	91
320	3.31	4000	20	6	0	0	78

321	3.68	3000	20	1	0	16	85
322	2.95	5000	7	1	1	3	91
323	2.60	3000	7	0	1	3	91
324	2.94	4000	18	0	3	6	111
325	3.24	10000	9	5	7	9	78
326	3.25	6000	24	11	7	10	96
327	2.42	4000	13	0	7	2	105
328	3.20	8000	20	5	8	18	81
329	2.90	6000	28	3	6	11	83
330	2.89	6000	13	3	7	18	88
331	2.89	4500	21	6	2	5	96
332	2.69	3000	33	12	19	27	76
333	2.68	3000	22	8	7	18	53
334	2.77	4000	11	5	4	5	94
335	2.95	3000	20	12	7	15	99
336	3.55	4000	22	12	7	18	53
337	2.60	3000	22	12	7	18	53
338	3.44	4000	22	0	7	18	53
339	3.44	4000	0	5	0	0	83
340	3.13	3500	22	3	7	11	75
341	3.17	6000	6	4	0	0	97
342	3.44	4000	16	14	0	0	104
343	3.05	10000	21	1	14	0	98
344	3.23	1500	18	3	7	6	60
345	3.14	4500	13	0	0	0	79
346	3.13	4000	16	1	0	0	105
347	2.80	5000	14	8	0	1	104
348	3.15	2800	15	1	7	20	73
349	3.18	3000	16	1	0	0	130
350	3.04	6000	19	2	3	3	86
351	3.31	6800	13	2	7	10	103
352	3.55	4000	15	3	0	1	112
353	3.54	8000	13	8	1	10	102
354	3.92	7000	18	3	11	27	61
355	3.75	6000	16	8	3	0	97
356	3.80	5000	18	4	11	27	60
357	3.50	3000	21	2	6	27	97
358	3.86	3000	13	0	1	0	92
359	3.78	4000	18	3	0	0	83
360	3.80	3000	9	6	2	2	83
361	3.74	7000	16	1	2	1	103
362	3.76	7000	15	8	2	1	93
363	3.75	10000	16	0	15	24	74
364	3.80	10000	10	1	0	0	80
365	3.40	9000	12	3	0	1	89
366	3.34	12000	2	3	0	0	100
367	3.47	4500	12	9	0	0	86

368	3.09	8000	20	5	12	15	79
369	3.44	9000	14	6	0	2	92
370	3.50	10000	12	6	4	1	91
371	3.57	10000	21	2	6	2	88
372	3.70	4000	30	0	5	6	74
373	3.34	4000	19	1	2	1	97
374	3.76	10000	20	7	3	16	96
375	3.77	5000	21	9	6	11	92
376	3.53	5000	21	2	0	4	92
377	3.81	5000	20	2	1	6	99
378	3.45	8000	19	1	3	15	101
379	2.30	3000	11	1	0	0	106
380	2.40	8000	19	5	2	2	114
381	3.40	4000	16	2	4	5	97
382	2.81	7000	14	5	2	17	89
383	2.66	4500	17	1	1	1	114
384	3.07	6000	16	1	1	2	92
385	3.65	5000	16	5	3	3	69
386	2.76	6000	12	5	2	4	69
387	2.47	4000	16	5	2	2	98
388	3.49	10000	18	1	4	16	86
389	2.80	7000	15	3	1	0	123
390	3.50	4000	20	0	1	4	109
391	3.57	5000	17	2	2	8	87
392	2.40	8000	13	9	0	0	110
393	2.80	5000	17	0	12	27	103
394	3.07	7000	0	0	0	0	101
395	3.25	8000	11	6	0	0	100
396	3.18	5000	12	1	4	1	94
397	3.03	2400	20	5	4	7	97
398	3.92	2000	18	1	1	1	114
399	3.08	1000	16	4	1	1	61
400	3.50	6500	12	5	4	15	103
401	3.33	3000	13	4	0	1	104
402	3.20	12000	22	3	7	4	99
403	2.80	20000	22	14	1	0	94
404	3.40	4000	18	2	7	27	78
405	3.71	5000	15	1	3	1	107
406	2.93	5000	21	1	3	7	92
407	3.14	4500	13	4	0	0	100
408	3.30	2000	22	0	7	4	99
409	3.73	4000	19	0	12	19	84
410	3.04	6000	13	8	0	1	100
411	3.12	1000	23	4	7	16	90
412	2.32	7000	15	0	1	8	95
413	2.97	20000	18	0	1	7	100
414	4.00	6500	16	0	0	1	100

415	3.77	10000	0	4	0	0	101
416	3.77	3500	11	2	1	0	94
417	2.25	6000	19	4	0	1	60
418	2.35	6000	15	1	4	7	105
419	3.93	6000	20	1	4	8	95
420	3.35	10000	20	2	1	1	100
421	3.30	3300	1	0	0	0	92
422	3.15	4000	0	4	0	0	110
423	3.56	3000	15	7	3	6	98
424	3.98	8000	21	3	10	11	83
425	2.85	2000	14	13	0	0	104
426	3.37	8500	20	10	10	27	83
427	2.50	5000	22	2	7	12	63
428	2.60	5000	18	7	2	6	108
429	3.00	10000	20	10	2	1	100
430	3.64	7000	25	11	6	1	116
431	3.43	5000	22	6	7	20	72
432	3.48	7000	25	8	1	6	104
433	3.30	5000	25	13	19	25	72
434	3.28	4000	20	3	17	27	84
435	3.32	3000	12	7	7	27	73
436	3.42	8000	20	1	14	27	75
437	2.67	10000	14	4	8	9	109
438	3.05	6000	19	4	10	18	100
439	2.59	6000	16	0	9	27	88
440	2.30	10000	13	1	7	11	88
441	3.33	4000	2	12	7	13	119
442	3.51	5000	26	5	21	27	70
443	2.08	3000	22	7	16	27	69
444	3.48	7000	29	2	12	20	113
445	3.10	6000	5	4	15	10	94
446	3.51	4000	23	3	15	27	89
447	3.25	6000	20	2	15	27	71
448	3.13	2000	18	7	9	12	97
449	2.67	10000	23	4	12	27	80
450	3.64	4000	20	2	7	9	104
451	3.06	4000	22	6	11	27	85
452	3.40	5000	13	8	10	16	121
453	3.55	3000	22	1	8	23	91
454	3.83	3000	16	9	9	16	95
455	3.37	3000	23	4	12	27	77
456	3.67	4000	23	0	7	12	98
457	3.09	3000	14	2	7	18	82
458	3.32	6000	19	14	9	15	115
459	3.30	5000	22	10	14	27	67
460	3.34	4000	23	4	21	27	74
461	3.08	4500	19	9	11	27	96

462	3.25	4000	24	7	17	27	79
463	3.55	3000	0	4	8	14	112
464	3.30	4000	16	1	11	24	104
465	3.00	5000	16	0	7	10	106
466	3.50	10000	19	6	7	9	90
467	3.36	9000	18	2	12	27	92
468	3.24	8000	17	0	10	27	98
469	3.45	8000	19	10	8	12	105
470	3.67	7000	19	3	8	27	103
471	3.00	15000	19	2	12	24	98
472	3.00	7000	20	6	8	20	109
473	3.25	10000	21	3	18	27	78
474	3.50	10000	19	0	12	24	98
475	3.23	5000	14	5	7	18	82
476	3.55	15000	22	1	10	27	97
477	2.99	9500	16	0	7	10	106
478	2.89	6500	14	7	8	23	107
479	3.25	5000	20	0	12	18	113
480	3.00	16000	13	12	7	11	88
481	2.99	7000	26	9	21	27	70
482	3.00	8000	23	2	12	27	77
483	3.00	7000	20	4	8	20	109
484	2.79	8000	19	1	10	18	100
485	3.08	8500	16	7	7	10	105
486	2.97	10000	23	2	12	27	78
487	3.25	8000	17	2	10	27	98
488	2.98	10000	20	4	8	20	103
489	2.75	6000	19	14	10	18	100
490	3.86	3000	22	6	14	27	67
491	3.97	3000	13	6	10	16	121
492	2.95	5000	13	0	10	16	121
493	3.54	7000	19	0	8	12	104
494	3.21	8000	9	4	7	9	108
495	3.11	8000	19	13	7	11	110
496	2.87	12000	29	1	14	27	66
497	3.00	9000	22	4	12	27	90
498	3.50	10000	20	15	7	27	101
499	2.98	6000	26	0	20	27	80
500	2.68	10000	21	14	9	15	101
501	3.22	8000	21	9	14	27	58
502	3.28	10000	20	12	14	27	78
503	3.23	8000	17	0	7	21	93
504	3.32	8000	16	0	7	9	124
505	3.05	6000	16	3	11	23	100
506	3.00	10000	21	0	17	27	65
507	2.40	5500	10	1	8	9	89
508	3.05	1000	17	4	12	19	104

509	2.98	3500	1	5	7	9	118
510	2.50	3000	19	5	16	27	91
511	3.65	5000	22	3	17	27	100
512	3.50	20000	21	4	11	27	98
513	3.41	10000	18	10	8	11	97
514	3.16	7000	24	5	15	27	88
515	3.57	5000	20	6	21	27	75
516	3.32	7000	19	16	14	27	71
517	3.17	4000	20	3	21	27	87
518	3.41	4000	24	6	12	20	111
519	2.69	6000	24	3	10	27	108
520	3.08	6000	15	3	12	27	71
521	3.45	10000	9	3	11	27	58
522	2.80	3000	19	1	12	27	92
523	3.50	6599	20	6	8	25	91
524	3.33	5000	12	6	9	9	95
525	2.95	8000	12	2	9	9	95
526	2.82	20000	29	5	12	22	38
527	2.99	10000	19	0	8	27	90
528	3.60	5000	14	2	8	27	62
529	3.03	8000	21	2	15	27	78
530	3.00	8000	21	1	9	18	112
531	3.67	5000	24	1	13	27	78
532	2.90	3000	19	7	10	20	87
533	3.32	6000	16	4	8	21	89
534	3.36	4000	20	5	21	27	78
535	2.83	6000	18	2	14	27	78
536	3.04	1000	13	8	8	9	99
537	2.69	6000	0	0	7	9	115
538	2.98	4000	0	0	7	25	78
539	3.03	7000	16	6	7	9	124
540	3.28	6000	19	3	17	27	79
541	2.79	4000	19	0	11	21	83
542	3.41	4000	20	6	11	22	100
543	2.57	4000	20	4	20	25	72
544	3.06	3000	20	3	10	27	92
545	3.02	14000	13	3	11	27	87
546	2.53	2000	23	5	10	27	83
547	3.18	18000	22	0	17	27	100
548	3.00	3000	0	3	7	25	78
549	3.53	5000	19	1	12	27	92
550	2.56	4000	24	5	13	27	78
551	3.30	5000	20	12	21	27	75
552	3.62	6000	21	3	18	27	88
553	3.31	5000	21	4	11	27	98
554	3.00	5000	19	3	8	11	97
555	3.03	8000	19	7	8	16	96

556	3.45	10000	18	1	11	21	104
557	3.09	3000	20	0	10	20	114
558	3.28	4000	0	0	7	18	38
559	3.08	6000	18	3	10	9	106
560	3.00	8000	16	4	10	25	104
561	2.95	3500	22	0	12	27	87
562	2.66	4000	0	4	7	18	38
563	2.75	3000	14	10	8	12	115
564	2.73	4000	27	0	17	27	67
565	3.69	5000	0	5	7	18	38
566	3.08	3000	18	2	11	27	109
567	2.54	4500	17	0	11	27	107
568	2.88	4500	19	0	7	9	126
569	2.54	4500	18	7	11	27	89
570	2.76	4000	12	0	20	27	74
571	2.76	7000	1	1	9	10	124
572	3.89	5000	21	3	7	9	97
573	2.64	10000	12	0	7	12	112
574	3.01	2500	19	3	11	27	89
575	3.51	3000	18	7	13	27	98
576	3.70	4000	20	8	8	19	103
577	3.82	10000	17	0	14	27	92
578	2.45	10000	20	9	7	17	108
579	3.70	7000	24	5	16	27	103
580	3.94	5000	19	5	17	27	84
581	3.62	6000	24	6	14	27	94
582	3.63	10000	27	6	13	27	89
583	3.67	7000	12	2	10	9	111
584	3.93	6500	12	3	9	22	99
585	3.73	10000	17	0	11	27	104
586	3.79	4500	18	3	11	27	89
587	3.62	7000	18	8	7	11	114
588	3.71	10000	16	3	14	27	107
589	3.70	8000	19	5	16	27	91
590	3.90	3000	21	0	13	27	98
591	3.57	10000	20	3	7	17	110
592	3.79	5000	20	9	7	25	94
593	3.81	5000	21	0	14	27	78
594	3.66	6000	0	3	7	9	79
595	3.73	6000	23	3	9	21	86
596	3.94	5000	21	2	9	20	77
597	3.72	3000	19	4	10	25	97
598	3.68	7000	13	8	7	27	116
599	3.56	8000	14	3	13	27	80
600	3.67	8000	8	2	8	27	119
601	3.72	5000	19	5	14	27	99
602	3.71	8000	20	12	13	27	83

603	3.70	3000	26	5	11	20	77
604	3.88	3000	27	12	7	14	124
605	3.93	3000	19	1	14	27	102
606	3.77	5000	21	4	14	16	88
607	3.49	42000	18	3	12	21	91
608	3.80	6500	19	8	12	27	93
609	2.32	3000	20	9	7	18	78
610	3.93	8000	18	1	4	5	93
611	3.87	15000	9	3	0	0	115
612	3.66	6000	0	12	0	6	99
613	3.79	5000	17	4	7	3	91
614	3.66	5000	23	7	5	19	69
615	3.72	4000	16	0	0	0	107
616	3.64	6000	18	2	0	0	101
617	3.87	4500	9	0	2	3	105
618	3.79	15000	18	2	3	2	102
619	3.79	4000	10	5	0	0	116
620	3.88	7000	12	8	10	27	80
621	3.81	6000	22	8	5	7	77
622	3.67	6000	23	1	13	25	77
623	2.40	3000	13	0	1	1	111
624	3.93	5000	25	15	0	0	87
625	3.56	3000	10	1	7	9	58
626	3.78	4000	12	11	7	7	92
627	3.72	7000	21	0	11	27	66
628	3.69	5000	13	0	0	0	96
629	3.73	6000	0	14	0	0	103
630	3.98	8000	20	14	1	0	58
631	3.83	10000	20	0	7	16	61
632	2.27	7000	15	13	1	1	90
633	3.63	10000	28	2	9	27	61
634	3.93	5000	17	0	5	5	92
635	3.93	4000	20	5	0	2	125
636	3.54	3000	15	3	2	3	102
637	3.66	6000	4	13	3	0	102
638	3.52	6000	24	2	18	27	63
639	3.30	6000	17	6	0	0	95
640	3.42	8000	20	6	7	15	104
641	3.64	10000	20	8	7	15	104
642	3.85	3500	18	0	8	27	85
643	3.90	5000	14	0	1	0	101
644	3.42	7500	17	7	1	1	103
645	3.02	7000	14	5	7	3	84
646	3.04	5000	17	1	4	3	100
647	3.78	3000	22	5	6	14	85
648	3.57	5000	17	9	4	3	100
649	3.64	4000	20	1	9	20	71

650	3.80	5000	16	3	1	0	72
651	3.04	2500	12	1	3	6	93
652	3.73	5000	14	10	0	0	98
653	3.05	7000	8	8	8	11	76
654	3.62	12000	12	0	6	7	92
655	3.62	12000	16	0	2	2	115
656	3.08	10000	11	5	0	1	102
657	3.50	5000	23	6	3	4	98
658	3.29	4000	18	5	8	20	98
659	3.00	5000	23	0	7	8	86
660	3.43	3500	14	5	4	3	92
661	3.77	10000	19	7	6	6	87
662	3.31	7000	24	5	7	27	76
663	3.31	7000	23	5	7	11	67
664	3.54	6000	23	11	7	8	84
665	2.43	6000	32	8	15	27	78
666	3.12	5000	19	7	6	10	73
667	3.76	4000	18	15	1	3	100
668	3.68	4000	27	10	11	27	69
669	3.58	10000	28	10	6	10	130
670	3.27	3000	28	10	6	10	102
671	3.13	5000	17	5	2	3	102
672	3.38	4000	19	2	4	18	95
673	3.53	10000	0	13	0	0	76
674	3.21	4000	24	4	11	27	66
675	3.56	5000	22	5	1	11	97
676	3.42	4000	20	1	7	11	58
677	3.49	4000	19	3	0	0	78
678	3.26	5000	25	1	4	10	85
679	2.17	6000	22	15	3	15	87
680	3.39	5000	0	10	14	18	58
681	3.29	4000	8	1	8	11	76
682	3.15	10000	15	1	8	5	100
683	3.30	4000	17	1	7	14	86
684	3.74	4000	1	1	0	0	120
685	3.08	6000	19	1	3	1	101
686	3.28	5000	19	1	0	0	78
687	3.59	4000	18	13	2	3	95
688	2.99	4000	19	4	6	17	58
689	3.10	5000	18	1	3	4	93
690	3.28	6000	16	5	1	0	67
691	3.49	4500	20	5	7	11	38
692	3.49	3000	18	2	1	2	74
693	2.48	3000	16	9	5	4	115
694	3.28	4300	10	1	0	8	79
695	3.89	3000	12	10	0	0	80
696	3.37	6000	10	3	3	2	116

697	3.63	3000	12	7	3	3	91
698	3.56	2000	14	4	4	2	89
699	3.48	4000	7	2	2	2	102
700	3.28	4300	18	4	1	1	103
701	3.12	3000	20	3	0	3	106
702	3.33	5000	20	8	7	11	66
703	3.70	3000	20	5	0	0	107
704	3.16	4800	12	3	4	3	110
705	3.18	3000	16	4	9	5	83
706	2.66	5000	7	9	2	2	102
707	3.74	6000	22	5	6	10	95
708	3.22	5000	19	8	4	6	92
709	2.72	6000	20	10	6	12	68
710	3.40	12000	30	6	21	14	96
711	3.25	5000	31	11	1	0	96
712	2.70	4000	20	9	8	11	59
713	2.42	4000	21	9	7	4	86
714	3.46	6000	21	4	7	4	86
715	2.64	5500	3	1	2	6	78
716	3.25	3500	17	5	2	1	99
717	4.00	6000	25	15	3	2	101
718	3.01	6000	20	0	0	8	78
719	2.66	8000	15	0	0	0	112
720	3.46	6000	9	9	0	0	107
721	3.22	5000	22	15	0	7	80
722	3.25	3500	21	2	7	8	78
723	3.25	5000	18	1	6	1	83
724	3.07	5000	15	10	0	4	115
725	3.44	6000	19	15	7	6	75
726	3.02	5000	22	4	5	5	113
727	2.20	6000	15	5	4	5	63
728	3.07	4000	20	4	10	8	69
729	2.01	4000	15	2	4	5	63
730	2.63	4000	9	4	4	7	100
731	2.70	4000	21	4	13	13	78
732	3.39	5000	21	4	0	4	79
733	3.06	4500	21	7	13	13	78
734	3.60	5000	3	0	4	3	122
735	3.12	5000	13	12	1	1	109
736	2.10	6000	31	1	10	8	86
737	3.38	6000	16	7	0	0	78
738	2.35	1300	14	1	0	1	107
739	3.06	4000	21	0	0	0	104
740	3.36	7000	19	2	0	0	107
741	3.00	5000	20	9	7	8	95
742	2.62	6000	17	5	7	11	83
743	2.68	12000	15	2	1	4	87

744	2.95	5000	17	2	3	1	108
745	3.62	6000	18	7	0	0	92
746	3.62	6000	19	1	8	14	83
747	2.20	3500	17	0	0	5	97
748	2.89	6000	11	3	1	0	106
749	2.68	3000	16	5	5	11	93
750	2.64	6000	14	8	4	9	78
751	2.50	6000	22	0	16	8	89
752	2.89	6000	18	2	6	6	95
753	3.07	6000	18	1	7	8	58
754	3.52	7000	18	16	2	3	99
755	3.07	6000	30	0	13	15	74
756	2.96	5000	0	0	0	0	78
757	2.58	5000	13	5	0	3	103
758	3.55	6500	26	2	9	10	98
759	3.30	10000	23	10	4	3	103
760	2.97	5000	22	12	0	0	102
761	2.60	6000	15	5	2	0	93
762	2.91	6000	16	2	8	8	78
763	3.39	6000	21	8	7	5	100
764	2.93	12000	24	7	1	2	103
765	3.52	6000	15	3	4	3	99
766	2.68	10000	18	6	3	3	109
767	2.40	10000	21	10	7	6	90
768	3.19	6000	24	7	1	0	97
769	2.98	7000	18	2	5	2	84
770	2.70	4000	15	0	2	2	114
771	2.56	5000	20	6	1	1	98
772	3.86	3000	16	4	4	5	85
773	3.65	4000	19	0	0	2	102
774	3.44	3000	10	0	0	2	110
775	3.76	3000	17	1	1	1	96
776	3.56	3000	21	8	5	5	98
777	2.85	4000	23	0	4	6	77
778	2.89	4000	17	7	0	0	109
779	3.07	3500	17	0	10	12	74
780	3.25	7000	0	2	1	1	117
781	3.15	6000	8	4	0	4	120
782	2.98	4000	19	2	3	7	80
783	2.77	8000	11	1	8	7	84
784	3.03	6000	12	3	3	1	88
785	2.98	9000	20	10	0	1	102
786	3.24	3500	19	12	3	0	103
787	3.12	3000	20	5	0	4	58
788	3.06	5000	28	1	5	7	94
789	3.14	3500	13	1	3	2	102
790	3.13	4500	20	0	3	1	98

791	3.13	4500	28	8	0	0	116
792	2.30	4500	20	3	4	1	112
793	3.31	6800	14	1	8	8	73
794	3.32	7000	16	8	2	2	78
795	3.15	6000	30	9	4	8	103
796	3.59	8000	23	0	21	13	75
797	2.82	6000	16	8	4	3	99
798	3.30	4000	22	6	5	8	76
799	3.14	3000	20	3	7	10	85
800	3.15	10000	21	8	7	8	99
801	3.21	4000	22	1	3	4	101
802	3.14	3000	17	1	3	3	82
803	2.81	3000	13	1	3	2	102
804	3.18	7000	17	5	6	7	81
805	3.36	4000	17	7	6	6	82
806	2.78	5000	26	5	3	5	92
807	3.12	6000	23	6	7	17	75
808	3.12	6000	13	8	0	1	102
809	3.23	4000	21	10	0	2	125
810	2.90	9000	20	8	7	8	82
811	3.23	6000	20	6	3	2	122
812	3.55	4000	24	5	2	5	90
813	3.15	2800	19	1	2	2	113
814	3.13	4000	21	4	8	0	96
815	3.19	3500	19	9	4	5	98
816	3.00	6000	19	9	3	1	82
817	2.64	1500	25	0	16	16	73
818	2.72	4000	17	5	0	0	95
819	3.27	5000	22	4	7	10	78
820	3.38	5000	13	9	1	3	96
821	3.75	7000	26	4	7	6	112
822	3.94	4000	27	9	11	9	78
823	3.93	3000	24	9	9	13	80
824	3.92	7000	25	3	14	13	83
825	3.62	4000	23	4	5	4	105
826	3.84	7000	25	1	7	5	104
827	3.76	10000	18	5	2	1	95
828	3.96	10000	22	6	5	8	106
829	3.74	10000	23	9	7	6	90
830	3.81	10000	22	1	8	13	70
831	3.76	7000	20	3	0	0	99
832	3.80	3000	18	13	0	0	87
833	3.59	5000	26	8	18	18	69
834	3.46	25000	25	7	6	4	105
835	3.30	8000	22	0	14	15	108
836	3.52	5000	18	7	0	0	105
837	3.24	15000	20	4	7	9	92

838	3.50	10000	23	16	3	5	86
839	3.50	8000	0	1	7	16	78
840	3.57	10000	20	4	0	1	78
841	3.64	20000	19	2	5	8	101
842	3.85	6000	18	9	0	1	102
843	3.52	5000	18	5	7	6	78
844	3.77	5000	18	8	4	6	68
845	3.79	7000	23	8	6	5	81
846	3.79	5000	30	8	19	18	75
847	3.62	5000	18	0	11	11	75
848	3.78	4000	16	6	0	0	104
849	3.72	4000	14	5	0	0	38
850	3.54	1000	12	4	0	0	98
851	2.53	6000	21	5	7	8	72
852	2.75	6000	21	0	7	8	78
853	3.45	3500	20	5	0	7	102
854	3.14	5000	26	5	0	0	123
855	2.98	4400	19	6	11	14	78
856	3.65	8000	21	2	3	8	97
857	3.50	10000	24	9	6	8	98
858	2.11	6000	27	8	8	7	85
859	2.74	5000	10	8	0	0	58
860	2.98	5000	19	5	7	8	82
861	2.81	5000	21	5	3	0	98
862	2.80	7000	14	8	0	1	104
863	3.44	6000	15	16	7	10	89
864	3.32	10000	35	12	19	22	66
865	3.40	10000	20	14	7	8	78
866	2.67	5000	20	0	3	10	89
867	3.30	2000	11	0	0	8	94
868	3.33	6000	13	11	1	0	106
869	3.20	3000	18	7	6	9	88
870	3.31	5000	13	10	5	4	82
871	3.28	4000	20	0	0	0	78
872	3.40	7500	20	10	0	7	102
873	4.00	15000	24	10	10	12	87
874	3.67	3000	25	0	10	8	96
875	3.79	3000	15	3	1	0	103
876	3.66	6000	23	5	3	3	77
877	2.49	6000	21	10	3	0	98
878	3.81	4000	22	6	9	10	83
879	3.81	4000	14	10	0	4	99
880	3.10	6000	9	2	4	7	99
881	3.90	6000	21	5	0	0	67
882	3.50	10000	22	10	3	1	111
883	3.02	4000	25	10	8	6	91
884	3.95	10000	11	8	2	8	58

885	3.66	6500	18	15	12	11	77
886	3.01	3000	19	9	9	21	59
887	3.15	17000	13	4	10	4	67
888	2.97	10000	20	8	3	1	92
889	2.47	22210	20	3	0	0	113
890	3.82	5000	21	4	2	0	80
891	3.27	2000	16	5	2	1	93
892	2.33	6000	27	1	10	16	86
893	3.36	7000	19	6	0	0	101
894	2.90	3000	22	1	5	6	94
895	3.00	6000	12	8	3	1	89
896	2.30	8000	19	6	12	13	82
897	2.82	4000	19	0	11	14	77
898	3.60	7000	16	1	1	0	124
899	2.33	6000	22	1	1	4	105
900	3.31	6000	15	1	0	0	86
901	3.40	5000	18	1	0	3	99
902	3.54	6000	20	1	3	0	115
903	3.53	6000	13	9	0	2	43
904	2.87	10000	26	15	7	6	112
905	3.39	6000	15	8	11	9	90
906	3.00	3000	19	9	7	8	82
907	3.72	4000	2	5	0	0	79
908	3.56	3500	21	4	9	9	87
909	3.85	4000	13	3	0	2	88
910	3.35	6000	17	4	5	2	101
911	3.29	4000	6	5	2	2	91
912	3.49	5000	2	2	3	4	98
913	3.50	9000	1	4	2	1	120
914	2.31	6000	3	6	4	0	102
915	2.43	6000	7	0	5	6	94
916	3.42	8000	0	0	0	0	115
917	2.99	5000	5	4	5	3	93
918	3.02	10000	10	4	0	0	119
919	3.37	20000	8	5	2	7	106
920	3.23	5000	8	0	5	8	110
921	3.21	6000	0	4	0	1	113
922	3.66	15000	9	3	3	0	102
923	3.42	10000	13	5	8	2	105
924	3.10	15000	8	5	5	5	105
925	3.00	8000	21	3	5	8	85
926	2.89	9000	0	0	0	0	113
927	3.33	9500	9	5	3	2	93
928	2.89	5000	16	4	9	8	107
929	2.98	8000	10	3	0	0	119
930	3.11	10000	7	1	4	7	109
931	2.67	5000	14	3	8	7	108

932	2.78	9000	7	0	6	0	101
933	3.01	5500	0	0	5	5	89
934	3.21	8000	10	0	7	6	107
935	2.56	7000	18	1	7	7	99
936	3.40	6000	12	1	6	6	113
937	3.36	60000	10	5	7	7	107
938	3.24	9500	14	6	6	9	107
939	3.40	6000	5	2	3	8	92
940	2.75	8000	11	4	6	5	101
941	3.45	12000	10	6	5	7	102
942	2.40	5500	13	0	6	8	93
943	3.00	6000	21	3	1	8	86
944	2.98	5000	20	0	0	1	92
945	3.22	8000	7	0	7	8	99
946	2.82	2000	12	3	7	8	94
947	3.48	4000	6	0	3	7	96
948	3.14	3000	7	0	5	8	85
949	3.78	6000	20	0	0	0	80
950	3.74	3000	13	0	4	2	103
951	3.87	5008	10	2	1	2	99
952	2.25	4000	18	1	1	0	99
953	3.25	10000	5	0	3	3	98
954	3.66	13000	0	8	3	2	96
955	2.96	5000	8	0	4	5	105
956	3.14	4000	13	0	5	5	104
957	2.74	5000	16	4	0	3	94
958	3.50	6000	15	0	5	8	113
959	3.00	5000	9	1	5	3	110
960	3.23	4000	20	1	7	0	78
961	3.86	3000	23	4	8	3	93
962	3.80	4000	12	0	9	8	95
963	3.20	6000	0	0	0	0	130
964	3.43	3000	9	4	5	3	110
965	2.78	3000	16	0	6	8	104
966	3.14	6000	15	6	7	1	107
967	3.62	20000	11	0	1	3	86
968	3.09	3000	11	0	5	7	110
969	3.26	10000	12	0	6	8	75
970	2.84	10000	16	5	3	4	83
971	2.83	4500	10	1	2	4	86
972	3.62	6000	19	0	2	0	81
973	3.28	4000	17	2	9	8	84
974	3.44	4000	0	0	1	3	109
975	3.02	5000	17	0	9	8	84
976	2.34	4000	17	1	9	8	78
977	3.76	4500	10	1	3	6	116
978	3.96	8000	2	6	5	0	125

979	3.71	10000	20	0	2	8	78
980	3.55	6800	10	0	2	4	79
981	3.00	6000	12	9	6	8	75
982	3.79	8000	22	9	10	9	79
983	3.10	10000	10	8	5	5	51
984	3.00	8000	3	1	3	6	106
985	2.57	6000	19	4	1	7	79
986	2.64	1500	16	11	0	0	92
987	2.88	5000	14	0	4	9	61
988	3.00	9000	12	5	6	10	75
989	3.48	4000	19	5	7	14	95
990	3.33	9500	16	8	9	9	107
991	3.00	10000	12	12	2	9	93
992	3.62	9000	28	1	21	27	85
993	2.56	7000	15	5	0	0	109
994	2.90	20000	19	4	1	0	104
995	3.20	7000	13	0	7	15	78
996	2.85	3500	21	14	3	13	96
997	2.63	4000	30	0	18	27	87
998	3.00	10000	22	6	0	14	98
999	2.97	6000	20	1	3	4	81
1000	2.72	4000	15	2	0	0	95
1001	2.50	4000	22	2	5	9	61
